# Defective repair of topoisomerase I induced chromosomal damage in Huntington’s disease

**DOI:** 10.1007/s00018-022-04204-6

**Published:** 2022-02-28

**Authors:** Nelma M. Palminha, Cleide Dos Santos Souza, Jon Griffin, Chunyan Liao, Laura Ferraiuolo, Sherif F. El-Khamisy

**Affiliations:** 1 School of Biosciences, Firth Court, Healthy Lifespan and Neuroscience Institute, University of Sheffield, Sheffield, UK; 2 Sheffield Institute for Translational Neuroscience (SITraN), University of Sheffield, Sheffield, UK; 3 Institute of Cancer Therapeutics, University of Bradford, Bradford, UK

**Keywords:** Huntington’s disease, DNA repair, TOP1cc, p62/SQSTM1, Chromatin ubiquitination, RNF168

## Abstract

Topoisomerase1 (TOP1)-mediated chromosomal breaks are endogenous sources of DNA damage that affect neuronal genome stability. Whether TOP1 DNA breaks are sources of genomic instability in Huntington’s disease (HD) is unknown. Here, we report defective 53BP1 recruitment in multiple HD cell models, including striatal neurons derived from HD patients. Defective 53BP1 recruitment is due to reduced H2A ubiquitination caused by the limited RNF168 activity. The reduced availability of RNF168 is caused by an increased interaction with p62, a protein involved in selective autophagy. Depletion of p62 or disruption of the interaction between RNAF168 and p62 was sufficient to restore 53BP1 enrichment and subsequent DNA repair in HD models, providing new opportunities for therapeutic interventions. These findings are reminiscent to what was described for p62 accumulation caused by *C9orf72* expansion in ALS/FTD and suggest a common mechanism by which protein aggregation perturb DNA repair signaling.

**Supplementary Information:**

The online version contains supplementary material available at 10.1007/s00018-022-04204-6.

## Introduction

Huntington’s disease (HD) is an autosomal dominant neurodegenerative disorder caused by expanded CAG repeats in exon 1 of the huntingtin gene (*HTT*, also called *IT15*), resulting in extended polyglutamine (polyQ) tracts in the N-terminal of the HTT protein [1]. Mutations in *HTT* gene are fully penetrant when CAG repeats go beyond a threshold of 40 [2]. The age of motor onset is tightly correlated with the length of the CAG tracts as longer expansions are associated with earlier onsets [3].

The nervous system, which is affected in HD, is constantly exposed to endogenous sources of DNA damage [4–6]. Neurons are highly metabolically active with a vast oxygen consumption, making them uniquely susceptible to lethal by-products that constantly threaten neuronal genome stability [7–9]. These endogenous genotoxins include the production of reactive oxygen species (ROS) and consequent stabilization of topoisomerase I (TOP1) cleavage complexes (ccs) due to the increased metabolic respiration and high transcription rates [7, 10, 11]. If unrepaired, these irreversible TOP1ccs represent a threat to neuronal genome stability since they interfere with transcription and can be turned into persistent chromosomal DNA breaks [10–13]. Accumulation of DNA double-strand breaks (DSBs) is harmful, ultimately causing neuronal death [14]. For protection, neurons developed extremely proficient DNA damage response mechanisms to defend them from these constant threats. It is not a surprise that defects in components of the DDR underpin the etiology of several neurodegenerative diseases [14–20]. For example, in ataxia telangiectasia (A-T), spinocerebellar ataxia with axonal neuropathy (SCAN1) and amyotrophic lateral sclerosis/ frontotemporal dementia (ALS/FTD) models, the defects in TOP1cc repair and consequent accumulation of DSBs over time were shown to trigger neuronal death [20–23]. Additionally, TOP1cc can also be trapped by camptothecin (CPT), a chemotherapeutic drug that specifically targets DNA-bound TOP1 [24]. Transcriptional TOP1cc-induced DSBs favor the repair by non-homologous end-joining (NHEJ) since these DSBs arise from broken DNA strands at close proximity [12, 25]. Additionally, previous studies have shown that CPT-induced DSBs activate ATM-mediated DDR, with a concomitant increase of γH2AX signal, H2A ubiquitination and 53BP1 recruitment in neurons [26, 27].

Impaired DNA repair is also involved in the pathogenesis of HD neurodegenerative disorders [28–32]. Abnormal activation of ATM signaling has been shown in several HD models [28, 33, 34]. HD murine brains, HD cells and post-mortem brain tissues from HD patients exhibit persistent γH2AX, indicating an accrual of unrepaired DSB [28, 35]. Additional to persistent ATM activation, HD cells also exhibit altered γH2AX dynamics, indicating a defect in the recognition and repair of DSBs [28, 35]. However, it is still unknown whether TOP1-related DNA lesions are also a source of DNA damage in HD. This is of particular interest given TOP1-mediated DNA breaks are common lesions that occur spontaneously in the brain [36].

Growing evidence supports a relationship between defects in autophagy and impaired DDR. Cells defective of autophagy mechanisms accumulate p62, a ubiquitin-binding protein that recognizes cargos carrying lysine 63 (K63)-polyubiquitin chains and recruits them to autophagosomes [37, 38]. Previous studies reported that as p62 accumulates in the nucleus of autophagy deficient cells, it binds and inhibits RNF168, an E3 ubiquitin ligase involved in DDR [22, 39]. RNF168 ubiquitinates K13 and K15 residues of histone H2A which signals for the recruitment of downstream repair proteins, including 53BP1 [39]. We have recently reported that ALS/FTD-*C9orf72* repeat expansions disturb ATM-mediated chromosomal-break repair due to accumulation of p62 and concomitant defects in H2A ubiquitination, resulting in increased DNA breaks and cell death [22]. However, whether and how these mechanisms operate in the context of HD remain unknown. Here, we report that TOP1-mediated DNA lesions are sources of DNA damage in HD. Moreover, expanded polyQ repeats drive cellular toxicity, at least in part, by promoting defects in DDR triggered by TOP1cc mediated DSBs due to the compromised activity of RNF168 that is caused by its increased interaction with p62.

## Materials and methods

### Cell lines

MRC5 and HEK293 cells were used for transient overexpression of HTT plasmids. The primary human skin fibroblasts, from unrelated individuals, GM08402 (Male, 32 yr, apparently healthy); GM04799 (Male, 47 yr, HD, 42 CAG) and GM04869 (Female, 32 yr, HD, 47 CAG) were obtained from Coriell Cell Repositories. The induced pluripotent stem cells (iPSCs) GM23225 (Female, 20 yr, HD, 68 CAG), obtained from Coriell Cell Repositories and the apparently healthy CS14 (Female, 30 yr) were used for differentiation into striatal neurons. Additional details about primary patient cell lines are provided in Table S1.

### Mammalian cell culture

MRC5 and HEK293 cells were grown in Dulbecco’s Minimum Essential Media (DMEM, Sigma-Aldrich) supplemented with 10% fetal bovine serum (FBS) 1% penicillin/streptomycin (Sigma-Aldrich) and 1% l-Glutamine (Sigma-Aldrich). Human primary fibroblasts were grown in DMEM (Sigma-Aldrich) supplemented with 10% fetal bovine serum (Biosera, FB-1001/500), 1% penicillin/streptomycin (Sigma- Aldrich), 1% l-Glutamine (Sigma-Aldrich) and 1% non-essential amino acids (Sigma-Aldrich).

### iPS Cell culture

Human iPSCs were maintained in Matrigel-coated plates according to the manufacturer’s recommendations in complete mTeSR™-Plus™ Medium. Cells were passaged every 6 days as clumps using ReLeSR™ an enzyme-free reagent for dissociation according to the manufacturer’s recommendations. For all the experiments in this study, iPSCs were used between passage 20 and 30, all iPSCs were cultured in 5% O_2_, 5% CO_2_ at 37 °C.

### Differentiation of iPSCs into neural progenitor cells (NPCs)

Neural differentiation of iPSCs was performed using the modified version dual SMAD inhibition protocol [40]. Briefly iPSCs were transferred for Matrigel-coated plate. On the day after plating (day 1), after the cells have reached ∼100% confluence, the cells were washed once with PBS and then the medium was replaced for neural medium (50% of KnockOut™ DMEM/F-12; 50% of Neurobasal; 0.5 × N2 supplement; 1 × Gibco® GlutaMAX™ Supplement; 0.5 × B-27, 50 U/mL penicillin and 50 mg/mL and streptomycin. The medium was additionally supplemented with SMAD inhibitors (DMH-1: 2 μM; SB431542:10 μM and CHIR99021: 3 μM)). The medium was replaced every day for 6 days. On day 7, the medium was replaced for neural medium supplemented with DMH-1:2 μM, SB431542: 10 μM and CHIR: 1 μM; All-Trans Retinoic Acid (RA): 0.1 μM and Purmorphamine (PMN): 0.5 μM. The cells were kept in this medium until day 12 when is possible to see a uniform neuroepithelial sheet. The cells were then split 1:3 with Accutase onto matrigel substrate in the presence of 10 µM of rock inhibitor (Y-27632 dihydrochloride), giving rise to a sheet of neural progenitor cells (NPC). In this stage the NPC were expanded in the same medium containing 3 µM CHIR99021; 2 µM SB431542; 0.1 µM RA; 0.5 µM PMN and 0.5 µM Valproic acid (expansion medium) and spit 1:3 once a week with Accutase. NPCs were frozen with expansion medium plus 10% DMSO in liquid nitrogen and cultured again in expansion medium after thawing.

### Differentiation of striatal neurons from NPCs

On day 0 NPCs were plated in Matrigel-coated six-well plates at a minimum density of 1 × 10^6^ cells/well. When cells reached 80% confluency the NPC cells incubated for 10 days in GABAergic 1–10 medium: Neural medium (as before) supplemented with 200 ng/mL of recombinant human SHH (PeproTech, Cat.: 100-45), 100 ng/mL of recombinant human DKK1 (PeproTech, Cat.: 100-45) and 30 ng/mL of recombinant human BDNF (ThermoFisher, Cat.: PHC7074). The media was changed every 2 days. At day 11 the cells were subjected to final passage. For the final passage the GABAergic progenitors were incubated with ROCK inhibitor (Y-27632 dihydrochloride, Tocris, Cat. No. 1254, 1:1000) for 1 h at 37 °C. The media was aspirated, and the cells were rinsed with PBS. The cells were then incubated with 1 mL Accutase™ (ThermoFisher, Cat.: A1110501) for 5–7 min at 37 °C. The cells were collected into a 15 mL tube containing twice the volume of GABAergic 1–10 medium. The cells were centrifuged at 200 rpm for 4 min, the supernatant was discarded and the cell pellet resuspended in 1 mL GABAergic 1–10 medium containing 10 μM ROCK inhibitor. The cells were then seeded in Matrigel-coated 96-well plates at a density of 2 × 10^4^ cells/well and incubated at 37 °C. After 24 h the medium was replaced with GABAergic 11–60 medium: Neurobasal™ medium supplemented with 1 × B27, 50 ng/mL BDNF and 1% penicillin/streptomycin. The medium was replaced every 2 days until day 60.

### Transfection

For immunofluorescence purposes, cells were transiently transfected with 500 ng of pEGFPC1-tagged plasmids containing the exon 1 of HTT with either 23 CAG repeats (GFP-Q23: wild-type HTT. Addgene, #40261) or 74 CAG repeats (GFP-Q74: mutant HTT. Addgene, # 40262). Transfection complexes were prepared in serum-free medium and Lipofectamine-2000 as transfection reagent at a ratio of 2.5:1 (Lipofectamine: DNA).

For western blotting, MRC5 and HEK293 cells plated in 10 cm^3^ dishes were transfected with 5 µg of GFP-Q23 or GFP-Q74. Transient transfection was obtained by using polyethylenimine at a concentration of 2:1 (PEI:DNA). After transfection, the cells were incubated for 48 h at 37 °C.

For RNF168 co-immunoprecipitation (Co-IP) assay, HEK293 cells were plated in 10 cm^3^ dishes. When around 80% confluent, the cells were transiently transfected with 5 μg of GFP-Q23 or GFP-Q74 plasmids. For Flag co-IP the HEK293 cells were seeded in 15 cm^3^ plates until 80%. The cells were co-transfected with 7.5 μg of pCDNA3.1-Flag-H2A K5-9-118-119-125-127-129R plasmid (Flag-H2A K13/K15; Addgene, # 63565) and 7.5 μg of GFP-Q23 or GFP-Q74. For both, the transfection complexes were prepared in serum-free medium and PEI at a ratio of 2:1 (PEI:DNA). The cells were incubated for 48 h at 37 °C.

For p62 siRNA experiments, MRC5 cells were seeded in 6-well plates at a cell density of 4.5 × 10^5^ cells/well. The following day, the cells were co-transfected with 2 µg of GFP-tagged plasmids (Q23 or Q74) using PEI as described above along with 25 nM of either p62 siRNA (Santa-Cruz Biotechnology, sc-29679) or scramble particles using DharmaFECT (Dharmacon™) at a ratio of 1:1 (v/v). Primary fibroblasts were seeded in six-well plates at a cell density of 2.5 × 10^5^ cells/well. After 24 h the cells were transfected with 15 nM of p62 or control siRNA particles using Lipofectamine RNAiMAX at a ratio of 1:3 (siRNA:RNAiMAX; v/v). The cells were then incubated for 48 h at 37 °C.

### Immunofluorescence

MRC5 and primary human fibroblasts were seeded in 24-well plates on coverslips at a density of 1 × 10^5^ and 3 × 10^4^ cells/well respectively. Forty-eight hours after transfecting MRC5 or 24 h after seeding human fibroblasts, the cells were treated with 10 μM CPT/DMSO or 0.5 μM CPT/DMSO respectively. To test γH2AX kinetics, primary human fibroblasts were treated with 2 μM CPT for 1 h, followed by CPT removal and incubation at 37 °C with CPT-free media for 1, 2, 4 and 24 h. For BrdU immunofluorescence, primary fibroblasts were incubated with 25 μM BrdU for 23 h; 10 μM CPT or DMSO was added 1 h prior to fixation. MRC5 cells were used as a positive control.

Cells were washed twice with cold PBS. The cells were then fixed in 10% formalin for 10 min at room temperature. The cells were washed twice with PBS and permeabilized for 5 min with 0.5% Triton X-100 followed by two washes with PBS. For BrdU immunfluoresence, cells were incubated with 2 M HCl for 20 min. This was removed, a PBS wash performed and residual HCl quenched with 100 mM sodium borate pH 8.5 for 5 min. Prior to incubation with primary antibody, the cells were blocked with 2% BSA for 30 min at room temperature. Next, the cells were incubated with primary antibody in 2% BSA for 1 h at room temperature. Cells were washed three times with PBS and incubated with Alexa Fluor® 488 or 594 goat anti-rabbit IgG secondary antibody (Life Technologies, 1:500 in 2% BSA), or Alexa Fluor® 568 goat anti-rat IgG secondary antibody (Abcam, 1:100 in 2% BSA) for 1 h at room temperature. Finally, cells were washed three times with PBS and mounted in glass slides using VECTASHIELD with DAPI (VECTOR Laboratories).

For TOP1cc immunofluorescence, primary fibroblasts were treated with 10 μM CPT for 10 min. Endogenous levels of TOP1cc were detected using this method in untreated cells. The cells were washed twice with PBS and fixed in 10% formalin for 15 min at 4 °C. After fixation, the cells were washed three times and permeabilized in 0.2% Triton X-100 for 2 min on ice, followed by incubation with 0.1% SDS for 5 min at room temperature. Blocking was carried out by incubating cells with TSM buffer (10% (w/v) skimmed milk; 150 mM NaCl; 10 mM Tris–HCl pH 7.4, in water) for 1 h. Next the cells were incubated with primary antibody (diluted in 5% goat serum: SLS, G9023-10ML) overnight at 4 °C. Next day, the cells were washed five times with wash buffer (0.1% BSA; 0.1% Triton X-100 in PBS) for 4 min each wash and incubated with Alexa Fluor® 488 or 594 secondary antibody (Life Technologies, 1:1000 in 5% goat serum) for 1 h at room temperature. Finally, cells were washed five times with wash buffer and mounted in glass slides as mentioned before.

For the striatal GABAergic neurons, immunofluorescence assays were performed in 96-well plates. The cells were washed with PBS and fixed with 4% PFA for 10 min at room temperature. After fixation, samples were washed three times with PBS and permeabilized with 0.3% Triton X-100 diluted in PBS for 5 min. The cells were subsequently blocked in 5% Donkey serum (DS) for 1 h. After blocking, cell cultures were incubated with the appropriate primary antibodies diluted in PBS containing 1% of DS overnight. Cells were then washed with PBS three times. Fluorescent secondary antibodies (Alexa Fluor 488, 555, 594 or 647, diluted 1:400 with DS) were subsequently added to the cells and incubated for 1 h. The samples were washed with PBS three more times and incubated with 1.0 mg/mL 4,6-diamidino-2-phenylindole (DAPI) for nuclear staining. All experiments included cultures where the primary antibodies were not added, non-specific staining was not observed in such negative controls.

The details for the primary antibodies used in this study are provided in Table 1.

**Table 1:** Primary antibodies used in this study

Antibody	Host species	Supplier (catalog no.)	Concentration	Application
53BP1	Rabbit	Bethyl (A300-272A)	1:1000	IF
TOP1cc	Mouse	Millipore (MABE1084)	1:1000	IF
γH2AX	Mouse	Merk (JBW301)	1:1000	IF
p62/SQSTM1	Rabbit	Merk (P0067)	1:1000	IF/WB
Beta III Tubulin (TUJ1)	Chicken	Merk (AB9354)	1:1000	IF
MAP2	Guinea pig	Synaptic Systems (188004)	1:1000	IF
Caspase 3, active (cleaved) form	Rabbit	Merk (AB3623)	1:200	IF
GABA	Rabbit	Sigma-Aldrich (A2052)	1:1000	IF
DARPP32	Rabbit	Abcam (ab40801)	1:100	IF
BrdU	Rat	Abcam (ab6326)	1:1000	IF
H2A	Rabbit	Abcam (ab18255)	1:1000	WB
RNF168	Mouse	Santa-Cruz Biotechnology (sc-101125)	1:1000	WB
Cleaved caspase-3 (Asp175, clone 5A1E)	Rabbit	Cell Signaling Technology (9664)	1:1000	WB
β actin	Mouse	Abcam (ab8224)	1:1000	WB

Details about host species, supplier, working concentration and application are provided

*IF* immunofluorescence; *WB* western blot

### Image acquisition and analysis

Immunofluorescence images from MRC5 and primary fibroblasts were obtained on a Leica FW4000 Fluorescent Microscope (Leica Microsystems) using the 63 × lens or Nikon confocal microscope system A1 (Nikon Instruments, Tokyo, Japan), using the 60 × lens. 53BP1 and γH2AX foci quantification in MRC5 and primary fibroblasts was done manually. Cells were considered positive if containing > 5 foci for 53BP1 staining and > 10 foci for γH2AX. TOP1cc foci quantification was conducted using ImageJ software.

Images from GABAergic neurons were acquired Opera Phenix™ High Content Screening System at 40 × magnification using the Harmony™ Image analysis system. We used 405, 488 and 594 nm and 647 lasers, along with the appropriate excitation and emission filters. These settings were kept consistent while taking images from all cultures.

### Cell lysis

For whole-cell lysate extraction, cells were lysed for 30 min on ice with 1% NP-40 lysis buffer (1% NP-40; 150 mM NaCl; 50 mM Tris pH 8) supplemented with 1 × protease inhibitor, 1 mM DTT and BaseMuncher, with periodical vortexing. The lysates were centrifuged at 13,200 rpm for 20 min and the supernatant was collected.

To extract chromatin-bound fractions, the cells were first incubated with hypotonic buffer (20 mM Hepes pH 8.0, 10 mM KCl, 1 mM MgCl_2_, 20% glycerol and 0.1% Triton-X-100) for 10 min on ice and centrifuged at 6400 rpm for 4 min, to remove the cytoplasmic fraction. Subsequently, to remove the nuclear soluble fractions, the pellets were resuspended in hypertonic buffer (20 mM Hepes pH 8.0, 1 mM EDTA, 20% glycerol, 400 mM NaCl and 0.1% Triton-X-100) and incubated for 20 min on ice with periodical agitation. The cells were centrifuged at 13,200 rpm for 5 min. To collect the chromatin-bound fractions, the remaining pellets were incubated with insoluble nuclear buffer (20 mM Hepes pH 8.0, 150 mM NaCl, 1% SDS, 1% NP-40 and 10 mM idioacetamide) for 50 min with constant agitation at 4 °C. The lysates were further incubated with BaseMuncher at 25 °C for 15 min and centrifuged at 13,200 rpm for 5 min.

### Co-immunoprecipitation (Co-IP)

#### RNF168 Co-IP

Magnetic Dynabeads™ protein G (Invitrogen) were first washed in PBS/0.01% Tween-20 (PBST) twice (30 µL beads/condition). The beads were then incubated for 1 h at room temperature with 2 μg RNF168 or mouse IgG antibody in PBST. The antibody was crosslinked to the beads using BS_3_ crosslinking agent (Thermofisher, #21580) according to the manufacturer’s protocol. The beads were then washed three times with PBST and equilibrated in 4 volumes/lysate of 1% NP-40 lysis buffer. 150–200 μg of nuclear extracts (combined soluble and insoluble nuclear fractions) were incubated with the crosslinked beads for 2 h at 4 °C. The beads were then washed once in 1% NP-40 lysis buffer, followed by three washes in 0.2% NP-40 lysis buffer. The beads were eluted twice in 20 μL 0.1 M citric acid pH 2.6 for 2 min each, at room temperature with mild agitation. The eluates were neutralized in 5 μL of 2 M Tris pH 8.

#### Flag Co-IP

Magnetic Dynabeads™ protein G (Invitrogen) were first washed in PBS/0.01% Tween-20 (PBST) twice (30µL beads/condition). The beads were then incubated for 1 h at room temperature with 5 μg Flag or mouse IgG antibody in PBST. The antibody was crosslinked to the beads using BS_3_ crosslinking agent (Thermofisher, #21580) according to the manufacturer’s protocol. The beads were then washed three times with PBST and equilibrated in 2 volumes/ diluted lysate of 1% NP-40 lysis buffer. One hundred micrograms of chromatin extracts were diluted 10 times in dilution buffer (50 mM Tris pH 8; 150 mM NaCl) and incubated with the crosslinked beads for 1.5 h at 4 °C. The beads were then washed twice in 1% NP-40 lysis buffer followed by two more washes in 0.2% NP-40 lysis buffer. The beads were eluted twice in 20 μL 0.1 M citric acid pH 2.6 for 5 min each, at room temperature with mild agitation. The eluates were neutralized in 5 μL of 2 M Tris pH 8.

### SDS-PAGE and western blotting

The lysates were mixed with 5 × SDS loading buffer, boiled at 95 °C for 5 min and run in 4–20% precast gel (Bio-Rad, USA) for 40 min-1 h at 180 V. The gel was transferred onto a nitrocellulose membrane (Trans-Blot® Turbo™ Blotting System). The membrane was blocked in 5% milk for 30 min and incubated overnight at 4 °C with primary antibodies diluted in 5% milk. The primary antibodies used for western blot are stated in Table 1. Next day the membranes were washed thrice with 1xTBST for 5 min and incubated with goat anti-rabbit or anti-mouse IgG (H + L) HRP conjugated (BioRad, 1:4000 in 5% milk) for 1 h. After three washes with 1xTBST, the membranes were revealed by exposure at a ChemiDoc™ (BioRad) imaging system using an enhanced ChemiLuminescence (ECL) substrate for detection. The images obtained were processed using Image Lab 4.1 software.

### Viability assay

Primary human fibroblasts were seeded in 96-well plates at a density of 5 × 10^3^/well. After 24 h the cells were exposed to CPT at concentrations between 0–10 μM for 96 h. Twenty microlitres of CellTiter® blue reagent (Promega) were added to each well and incubated overnight at 37 °C. The fluorescence was recorded at 540/590 nm using a microplate reader (FLUOstar Omega, BMG Labtech).

### Neutral comet assay

Primary human fibroblasts were seeded in six well plates at a density of 1 × 10^5^/well. After 24 h the cells were exposed to 60 μM CPT for 30 min at 37 °C. The media was replaced with fresh media and cells allowed to recover for 0–4 h (timepoints are indicated in the relevant figures). Cells were trypsinised then resuspended in 0.8% low melting temperature agarose. This was layered on top of pre-prepared 0.8% normal melting point agarose on glass slides. Cells were lysed overnight at 4 °C in lysis buffer (2.5 M NaCl, 10 mM Tris–HCl, 100 mM EDTA, 1% Triton X-100, 1% DMSO). The slides were immersed in electrophoresis buffer (300 mM sodium acetate, 100 mM Tris–HCl, 1% DMSO) and allowed to equilibrate. The same buffer was then used for electrophoresis (25 V for 1 h at 4 °C). Slides were stored at 4 °C in 0.4 M Tris–HCl pH 7.4 for 24–48 h prior to imaging. Cells were stained with SYBR Gold (1:10,000) and imaged using a Nikon Eclipse TE300 inverted microscope. Comets were scored using Comet Assay IV. At least 100 comets were scored per sample.

### Statistical analysis

All graphs and statistical analysis were generated using GraphPad Prim (GraphPad Software Inc.). All data are presented as mean ± s.e.m. Student’s *t*-test was used to compare the means between two groups. Comparisons between 3 or more groups were performed using One-way ANOVA, followed by post hoc Tukey’s test for multiple comparisons. Area under the curve (A.U.C.) was calculated using the Prism 9 integrated formula, considering baseline as Y = 0 (GraphPad Statistics Guide/AUC). Statistical significance was considered when *P* < 0.05.

## Results

### Expression of mutant huntingtin (HTT) causes defective 53BP1 recruitment

**Fig. 1:**
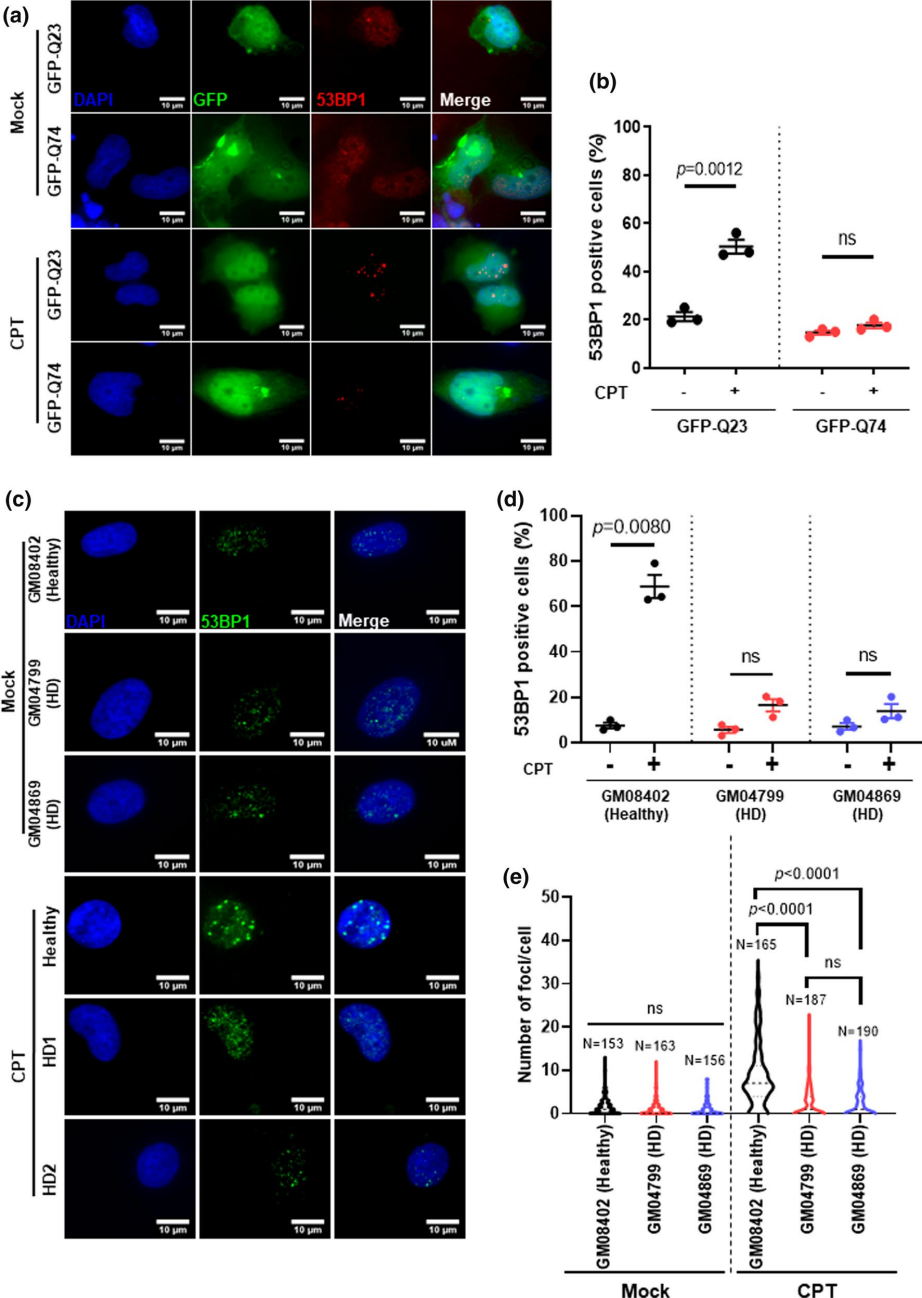
Expression of mutant huntingtin drives deficient 53BP1 recruitment in response to DNA damage. MRC5 cells transiently transfected with HTT GFP-tagged plasmids containing either 23 CAG repeats (GFP-Q23: wild-type HTT) or 74 CAG repeats (GFP-Q74: mutant HTT). Cells were treated with either DMSO or 10 µM CPT for 1 h. **a** Representative images of MRC5 immunostained with 53BP1 are shown (scale bar: 10 µm). **b** The percentage 53BP1 positive cells (> 5 foci) was quantified and analyzed using Student’s *t*-test (*n* = 3, 50 cells per replicate). Error bars represent ± s.e.m. **c**–**e** GM08402 represents the unaffected individual (healthy). GM04799 (HD: 42 CAG) and GM04869 (HD: 47 CAG) are fibroblasts retrieved from patients clinically affected with Huntington’s disease (HD). All cells were purchased from Coriell Institute. **c** Representative image showing primary human fibroblasts immunostained with 53BP1 after treatment with 0.5 µM of CPT for 1 h (scale bar: 10 µm). **d** The percentage average of 53BP1 positive cells (> 5 foci) was quantified and analyzed using Student’s *t*-test (*n* = 3, 10 fields per replicate). **e** Violin-plot showing the number of 53BP1 foci per cell, quantified from three biological experiments and analyzed using Kruskal–Wallis test (10 fields per replicate, ± s.e.m.). Error bars represent ± s.e.m. *ns* nonsignificant

Cells expressing exon 1 of the *HTT* gene with expanded CAG repeats were sufficiently able to mimic the phenotypes seen in HD cells [41]. To examine the effects of mutant HTT in the repair of DNA breaks induced by TOP1, we exposed MRC5 cells transiently expressing a GFP-fusion plasmid containing the exon 1 of *HTT* with either 23 CAG repeats (GFP-Q23, wild-type HTT) or 74 CAG repeats (GFP-Q74, mutant HTT) to 10 µM of the TOP1 inhibitor, camptothecin ‘CPT’. Immunostaining with an anti-53BP1 antibody revealed an increase in the percentage of GFP-Q23 cells positive for 53BP1 after treatment with CPT in comparison with mock (DMSO) treated cells. However, GFP-Q74 expressing cells failed to respond to CPT treatment, showing no increase in 53BP1 foci (Fig. 1a, b). We next investigated 53BP1 foci formation after DNA damage in primary fibroblasts from a healthy individual (Healthy: GM08402) and from two HD patients; HD1: GM04799; HD2: GM04869 [42–44]. HD primary fibroblasts exhibited reduced percentage of 53BP1 positive cells in comparison to healthy controls, after DNA damage (~ 20% vs ~ 60%, respectively) (Fig. 1c, d). HD1 and HD2 patient fibroblasts also displayed fewer number of 53BP1 foci per cell after CPT treatment (HD1, 2.5 ± 0.25; HD2, 2.3 ± 0.23 foci per cell) in comparison with healthy fibroblasts (Healthy, 8.5 ± 0.56 foci per cell) (Fig. 1e). We confirmed that most (> 80%) of the primary fibroblast cells were in G1 phase using BrdU incorporation and immunofluorescence, and that there was no difference in G1 phase fraction between healthy and HD cells or between DMSO and CPT treatment conditions (Fig. S1a and b). Furthermore, we used a resampling strategy (see supplementary methods) to confirm that the low proportion of S phase cells did not impact upon our observation of deficient 53BP1 recruitment in CPT-treated HD cells (Fig. S1c). These results suggest that mutant HTT compromises the recruitment of 53BP1 into repair foci and that most 53BP1 activity is directed towards G1 phase damage.

### HD patient fibroblasts accumulate TOP1 cleavage complexes

53BP1 recruitment to the chromatin is regulated by phosphorylation events set out by the master kinase ATM [45]. Additionally, 53BP1 also sustains ATM retention at the chromatin, suggesting a feedback loop in which 53BP1 and ATM cooperate to maintain the DDR in response to DSBs [17, 46]. A previous study has suggested that mutant HTT promotes cytoplasmic sequestration of ATM, preventing its nuclear translocation during DSB repair [35]. Another report showed that HTT participates in the oxidative damage response in an ATM-dependent manner, a mechanism found to be disrupted by mutant HTT as demonstrated by increased oxidative DNA damage in HD cells [47]. It is therefore possible that ATM:HTT complex participates in DDR induced by other genotoxins, including TOP1cc-induced damage. Additionally, since mutant HTT also interacts with nuclear pATM, it would be of interest to examine whether and how this interaction interferes with the normal function of ATM.

**Fig. 2:**
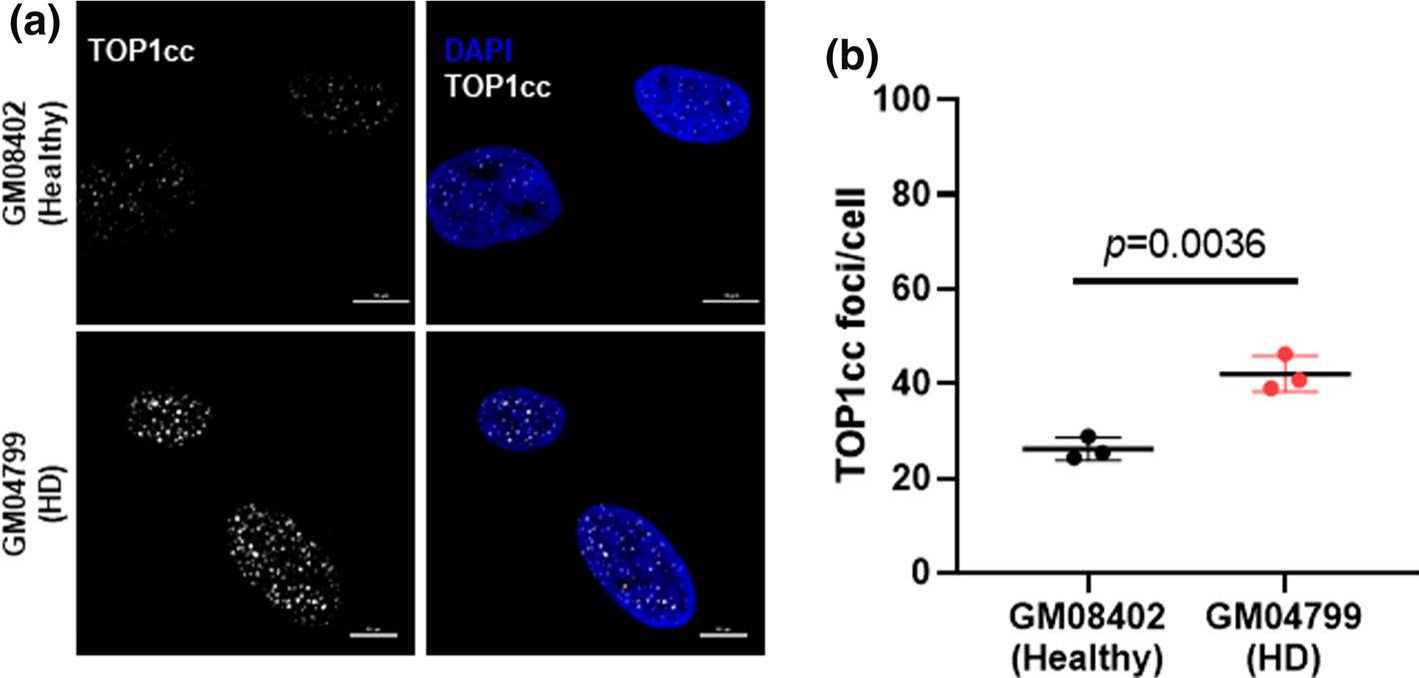
HD patient-derived fibroblasts show increased TOP1cc levels after CPT treatment. **a** Representative images of primary skin fibroblasts from a healthy individual (GM08402) and a HD patient (GM04799, 42 CAG) after treatment with 10 μM CPT for 10 min and immunostained with a specific antibody against TOP1cc. Scale bar: 10 μm. **b** The number of TOP1cc foci per cell was quantified and analyzed by Student’s *t*-test. Data are presented as average of three independent experiments ± s.e.m

Previous studies have demonstrated that ATM deficiency is associated with TOP1cc accumulation in the brain [11, 21, 48]. TOP1ccs are endogenous events that occur spontaneously in the brain [36] and can also arise from exposure to CPT and its derivates [24, 49]. Trapping of TOP1cc precedes the formation of TOP1-mediated DNA DSBs [12]. In line with these studies, we hypothesized that if binding of mutant HTT incites defects in ATM signaling, then HD cells would exhibit higher levels of TOP1cc after exposure to CPT. To test this, we treated healthy (GM08402) and HD (GM04799) fibroblasts with 10 μM CPT for 10 min and visualized TOP1cc by immunofluorescence (Fig. 2a). Immunostaining with a specific TOP1cc antibody showed that after CPT treatment, HD fibroblasts exhibited a higher number of TOP1cc foci/cell in comparison with healthy GM08402 fibroblasts (HD: 42.1 ± 3.8 foci/cell; Healthy: 26.2 ± 2.4) (Fig. 2b). Complementary analysis of the endogenous levels of TOP1cc in untreated fibroblasts also indicate a significant accrual in the number of TOP1cc foci/cell in GM04799 HD fibroblasts in comparison with GM08402 (HD: 11.9 ± 0.1; Healthy: 8.9 ± 0.2) (Fig. S2a and b).These results suggest an increased accumulation of TOP1cc in HD cells, in agreement to what was observed in ATM deficient models [21].

### Accumulation of unrepaired DNA damage compromises cell viability in HD patient fibroblasts

**Fig. 3:**
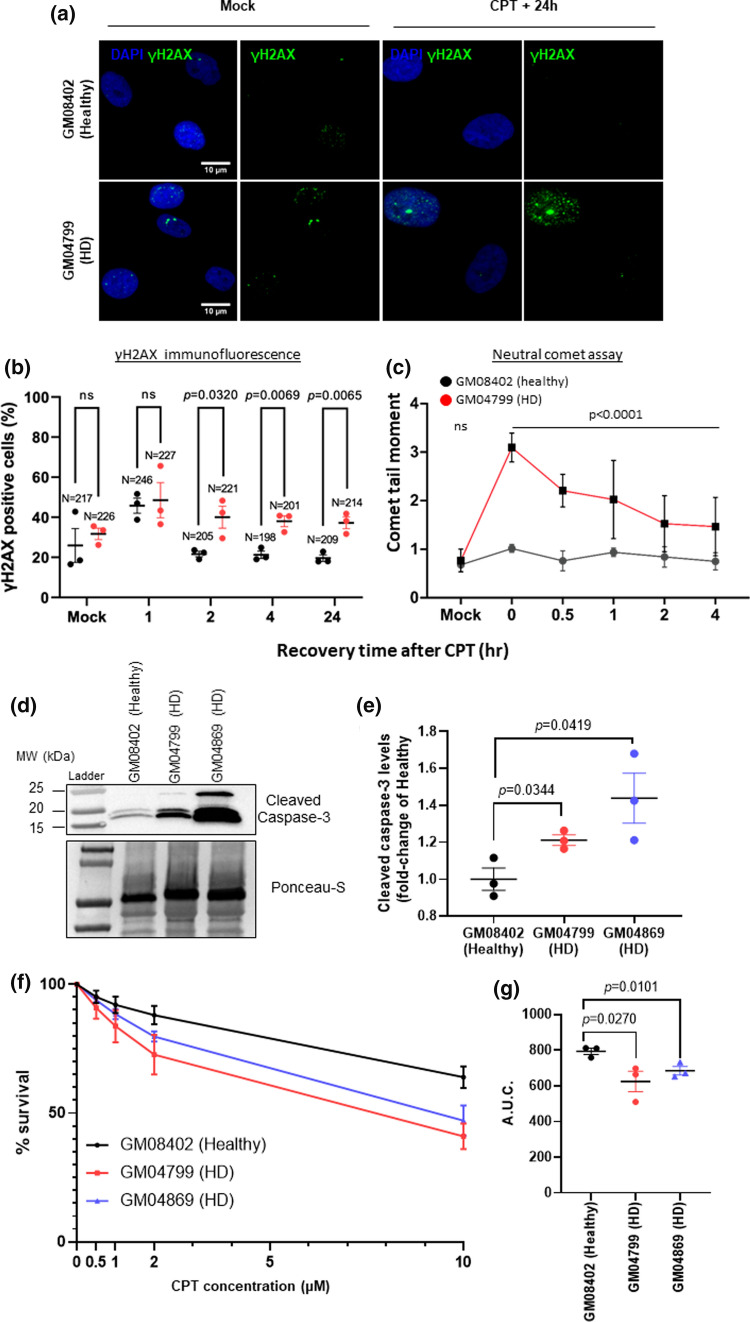
Accumulation of DNA damage compromises cell viability in HD fibroblasts. **a** Representative images of primary fibroblasts immunostained (Healthy: GM08402; HD: GM04799, 42 CAG) with an anti-γH2AX antibody after treatment with 2 μM CPT for 1 h and recovery in complete medium for 1–24 h (scale bar: 10 µm). **b** The percentage (%) of γH2AX positive cells (> 10 foci) was quantified for each time point and analyzed by Student’s *t*-test. Data are shown as average of three independent experiments (10 fields each; *N* = total number of cells counted). Error bars: ± s.e.m. **c** Healthy (GM08402) or HD (GM04799) fibroblasts were treated with 60 µM CPT for 30 min then allowed to recover in CPT-free media for 30 min, 1 h, 2 h and 4 h. Double-strand breaks were measured by neutral comet assays and the mean comet tail moments from three independent experiments analyzed by one-way ANOVA with Dunnett’s multiple comparisons test using the healthy fibroblast data as the comparator. **d** Western blotting analysis of fibroblasts after treatment with 10 μM CPT for 72 h and incubation with a cleaved caspase-3 specific antibody. Ponceau for loading control. **e** Levels of cleaved caspase-3 normalized against Ponceau. Fold-change relative to healthy cells was calculated and analyzed by Student’s *t*-test. Error bars: ± s.e.m; *n* = 3. **f** Sensitivity to CPT treatment of healthy and HD patient fibroblasts was monitored by CellTiter-Blue® after 96 h treatment with CPT (0–10 µM). *Y*-axis represent the mean percentage survival (% survival) plotted against CPT concentration (µM) (*n* = 3). % survival in treated fibroblasts was assessed by normalizing against the corresponding untreated condition (0 µM). Error bars represent ± s.e.m. **g** The area under the curve (A.U.C.) was calculated and plotted as average of three biological replicates. The data were analyzed by Student’s *t*-test (error bars: ± s.e.m.). *ns* nonsignificant

To analyze whether the defect in 53BP1 and the increase in TOP1cc in HD cells culminated in defective DNA repair and consequent accumulation of DSBs we next examined DSB repair kinetics following CPT treatment. The cells were treated for 1 h with 2 μM CPT, followed by removal of CPT and recovery in complete media for 1–24 h. The cells were then subjected to immunofluorescence analysis of the DSB marker, γH2AX (Fig. 3a). Both healthy and HD cells exhibited a peak on the percentage of γH2AX positive cells after 1 h recovery from CPT treatment (Fig. 3b), which is consistent with previous reports showing that CPT activates ATM and induces γH2AX in non-cycling cells. However, in the subsequent post-CPT treatment time points, the healthy fibroblasts experienced a decrease in the percentage of γH2AX positive cells, to levels close to those of the mock treated cells, whereas HD cells did not. Although the percentage of γH2AX positive cells at 2 h, 4 h and 24 h post CPT treatment decreased in HD cells compared to the 1-h time-point, levels remained significantly higher than the healthy cell line (Fig. 3b). We confirmed that the increase in γH2AX foci in HD cells was directly related to DNA damage by neutral comet assays, which measure double strand breaks. Cells were treated with CPT and comet tail moment was recorded during a recovery time course. A higher level of DNA damage was observed in HD cells compared to healthy controls immediately after CPT treatment. In addition, the mean comet tail moment remained higher in HD cells 1–2 h after recovery from CPT treatment (Fig. 3c and Fig. S3a and b). Together, these results suggest that HD cells display a slower repair of DNA damage induced by CPT.

Next, we tested if the defective DNA damage repair observed in HD cells in response to CPT causes increased sensitivity and cell death. We examined the expression levels of cleaved caspase 3, a maker for active execution of apoptosis after treatment with CPT for 72 h (Fig. 3d). Western blotting analysis showed increased caspase 3 activation in both HD patient fibroblasts in comparison with healthy cells (Fig. 3e). If DNA damage is left unresolved, cells undergo cell cycle arrest and apoptosis. Consistently, cell viability experiments revealed that both patient-derived HD cells displayed decreased survival in response to increasing CPT concentrations, in comparison with healthy control cells (Fig. 3f, g). Together, these findings suggest that HD cells are hypersensitive to CPT.

### Striatal neurons from HD patients exhibit decreased 53BP1 signaling and increased activation of apoptotic markers after topoisomerase-induced DNA damage

HD is characterized by the progressive degeneration of the striatum [1, 50]. The gamma-aminobutyric acid (GABA)ergic medium spiny neurons (MSNs), which comprise around 95% of the striatal neuron population, are particularly vulnerable to the toxic effects of mutant HTT, where loss of MSNs is observed during the progression of HD [51, 52]. Thus, we next tested whether the findings observed in non-neuronal cells are also exhibited in GABAergic striatal neurons. We differentiated neural progenitor cells (NPCs) derived from induced pluripotent stem cells (iPSCs) from an HD patient (GM23225) and a healthy individual (CS14) into striatal GABAergic neurons using a three-step differentiation protocol (Fig. S4) [53].

**Fig. 4:**
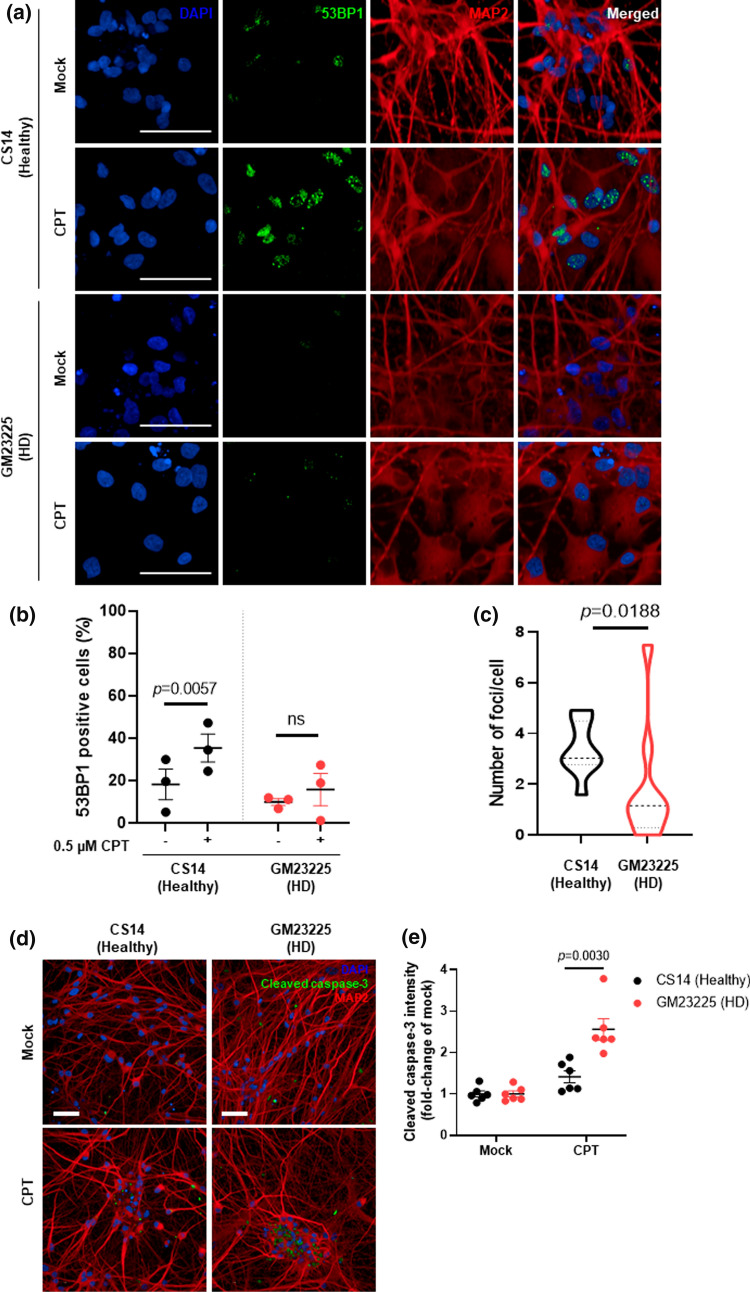
GABAergic neurons from HD patients exhibit decreased 53BP1 signaling and increased activation of the apoptotic marker cleaved caspase-3. **a** Representative images of GABAergic neurons differentiated from NPCs from a healthy individual (CS14) and a patient with HD (GM23225, 68 CAG) immunostained with an anti-53BP1 antibody after treatment with 0.5 μM CPT or mock (DMSO) for 1 h (scale bar: 50 µm). DAPI shows nuclear staining. MAP2 is a specific marker for neuronal cytoskeleton. **b** The percentage (%) of 53BP1 positive cells (> 2 foci) was quantified for each time point and analyzed by Student’s *t*-test. Data are shown as average of three independent experiments. Error bars represent ± s.e.m. **c** Violin-plot showing the number of 53BP1 foci per cell after treatment with 0.5 μM CPT. **d** Representative images of GABAergic neurons differentiated from NPCs immunostained with a specific cleaved caspase-3 antibody after treatment with 10 μM CPT or mock (DMSO) for 48 h (scale bar: 50 µm). **e** The intensity of cleaved caspase-3 signal was quantified and data are shown as average of six technical replicates across two biological experiments and was analyzed by Student’s *t*-test. Error bars represent ± s.e.m. *ns* nonsignificant

After treatment with CPT, while healthy neurons showed a significant increase in the percentage of cells positive for 53BP1 staining, striatal neurons from HD patients exhibited almost no 53BP1 response (Fig. 4a, b). Further analysis revealed that healthy neurons scored 3.4 ± 1.1 53BP1 foci/cell after CPT treatment, while HD patient striatal neurons only scored 1.8 ± 2.4 foci/cell (Fig. 4c). These results are in line with observations in non-neuronal cells and indicate that HD striatal neurons also failed to respond to DNA damage induced by CPT.

We next tested whether treatment with CPT also induced the activation of the apoptotic marker, cleaved caspase 3, in HD GABAergic neurons (Fig. 4d). HD neurons exhibited a ~ 2.6-fold increase in the levels of cleaved caspase 3, compared with ~ 1.4-fold increase in the healthy neurons. Together, these results suggest HD neurons have increased sensitivity to CPT as illustrated by the increased levels of apoptotic cell death, similar to what was observed in HD fibroblasts.

### Expression of mutant huntingtin (HTT) impairs histone H2A ubiquitination

**Fig. 5:**
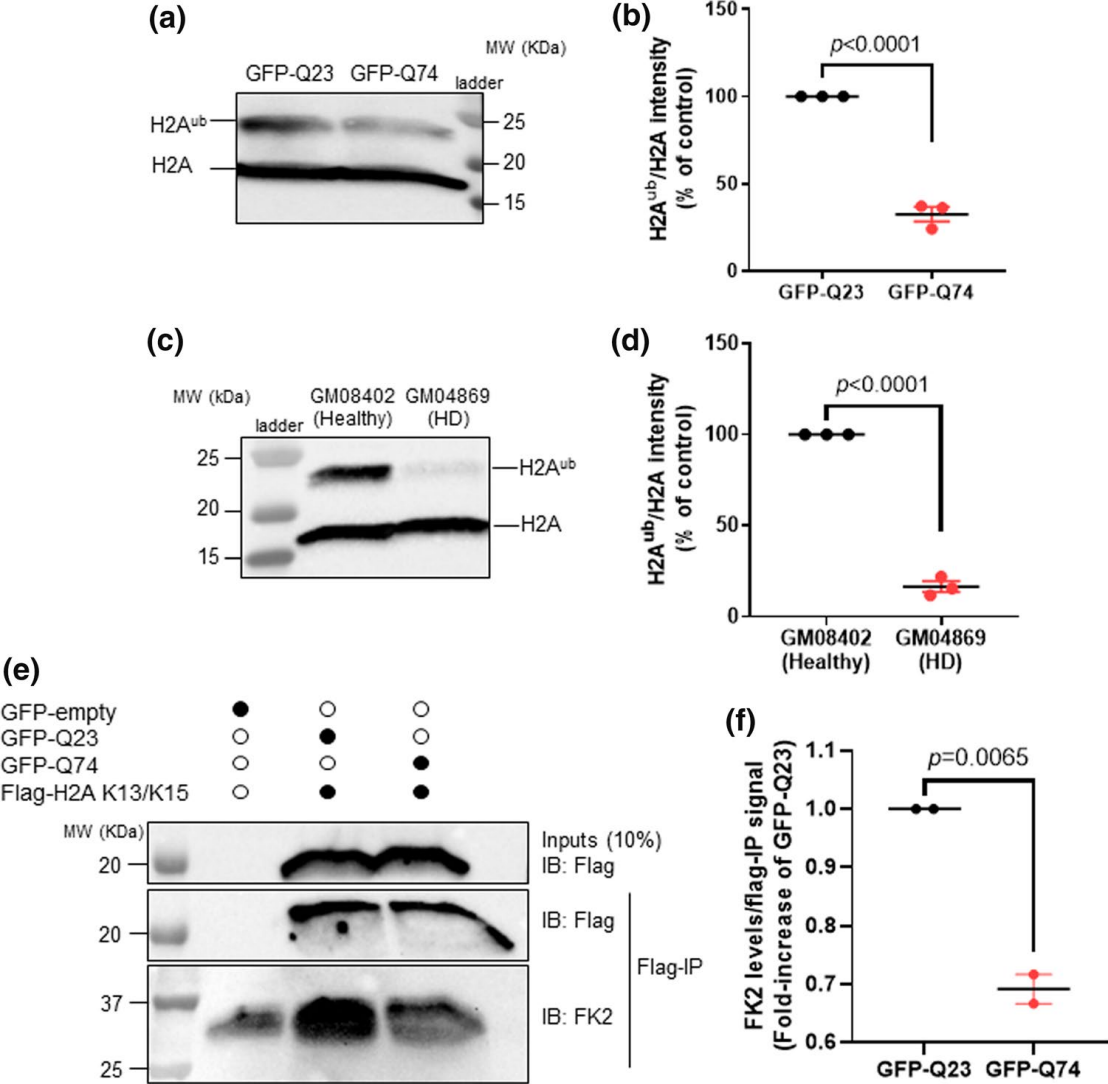
Expression of mutant huntingtin impairs H2A ubiquitylation. **a** Western blotting of chromatin-bound fractions from MRC5 overexpressed with GFP-Q23 or GFP-Q74 after probing with an antibody against H2A. Bottom band at ~ 17 KDa represents unmodified H2A, showing equal loading. Top band represents ubiquitinated H2A (H2A^ub^). **b** Levels of H2A^ub^, normalized against H2A and presented as percentage (%) of healthy and analyzed by Student’s *t*-test. (Error bars: ± s.e.m, *n* = 3). **c** Chromatin-bound fractions from healthy (GM08402) and HD (GM04869, 47 CAG) fibroblasts were analyzed by western blotting. Bottom band represents unmodified H2A. Top band represents H2A^ub^. **d** Levels of H2A^ub^, normalized against H2A and presented as % of healthy cells. The data were analyzed by Student’s *t*-test and is shown as means ± s.e.m. (*n* = 3). **e** Chromatin-fractions of HEK293 co-transfected with GFP-Q23 or GFP-Q74 and Flag-H2A K5-9-118-119-125-127-129R mutant plasmids (Flag-H2A K13/K15) were subjected to Flag immunoprecipitation (Flag IP) after treatment with 10 μM CPT for 1 h. GFP-empty represents negative control. Flag-H2A K13/15 ubiquitination was detected using a pan-ubiquitin antibody (Fk2). 10% of the lysates were analyzed by Western blotting. GFP-Q23 and GFP-Q74 express similar levels of Flag-H2A K13/K15 (Inputs). **f** Levels of Fk2 were quantified and normalized against the corresponding Flag-IP signal. Data show means ± s.e.m. (*n* = 2) and were analyzed by Student’s *t*-test

53BP1 recruitment to DSBs depends on histone H2A ubiquitination events. RNF168 is an E3 ubiquitin ligase that catalyzes ubiquitination of H2A [54, 55]. Recognition of ubiquitinated H2A by 53BP1 promotes its enrichment at damaged chromatin and subsequent DSB repair [56]. We thus tested whether expression of mutant HTT impairs H2A ubiquitination. Since our data indicate that ectopic expression of the CAG expansions in MRC5 cells was able to recapitulate DNA repair defects and cellular toxicity observed in HD GABAergic neurons, we decided to perform the biochemical experiments using this model. Chromatin-bound fractions from MRC5 cells expressing GFP-Q23 and GFP-Q74 were analyzed by Western blotting using an antibody against H2A, which recognizes not only unmodified H2A (~ 17KDa) but also ubiquitinated forms of H2A (Fig. 5a). Notably, expression of mutant HTT caused a decrease in H2A ubiquitination by ~ threefold in comparison with expression of wild-type HTT (Fig. 5b). Consistent with these results, Western blotting analysis of primary fibroblasts from HD GM04869 patient also showed a ~ sixfold reduction in H2A ubiquitinated species in comparison with healthy GM08402 fibroblasts (Fig. 5c, d).

In both the MRC5 and primary fibroblast models, CPT treatment restored global ubiquitination of H2A (Fig. S5a and b). We therefore asked if specific H2A residues were ubiquitinated after CPT induced DNA damage. Anchoring and stabilization of 53BP1 at the chromatin during DNA repair depends on the recognition of the damage-induced H2AK13/15^ub^ by its UDR motif, and binding to H4K20^me2^ by its Tudor domain [57–59]. Therefore, we investigated the ubiquitination levels of histone H2A, specifically at K13 and K15 in cells ectopically expressing the CAG expansions. Cells were transfected with either GFP-Q23 or GFP-Q74, together with a Flag-H2A plasmid in which all known lysine ubiquitination sites were mutated to arginine except K13 and K15 (Flag-H2A K13/K15). This way, we were able to specifically analyze the modifications occurring at K13/K15 of H2A. These experiments required double transfections and large amounts of cell lysate, which was not feasible in transiently transfected MRC5 cells. Therefore, we used HEK293 cells, which have been used by other labs to study the effects of ectopically expressing mutant HTT [60]. After treatment with CPT, the chromatin fraction was extracted and incubated with magnetic beads coated with a Flag antibody. The eluates were then subjected to Western blotting analysis, using a pan-ubiquitin antibody (FK2). Cells that express GFP-Q23 exhibited more H2AK13/K15 ubiquitination in comparison to cells expressing mutant HTT (Fig. 5e, f). To note, although ubiquitin seems to stick nonspecifically to the magnetic beads, as observed in the first lane, the strong Fk2 signal in the second lane indicates the majority of the ubiquitination observed was specific to Flag-H2A K13/K15 (Fig. 5e). Together, these results suggest defective H2A ubiquitination in HD models. Whilst global H2A ubiquitination was restored by CPT treatment, a specific defect of H2AK13/15 ubiquitination remained in HD cells after CPT-induced DNA damage. This may explain the defective 53BP1 recruitment.

### p62 depletion restores 53BP1 signaling and reduces hypersensitivity to topoisomerase I DNA damage in HD cells

**Fig. 6:**
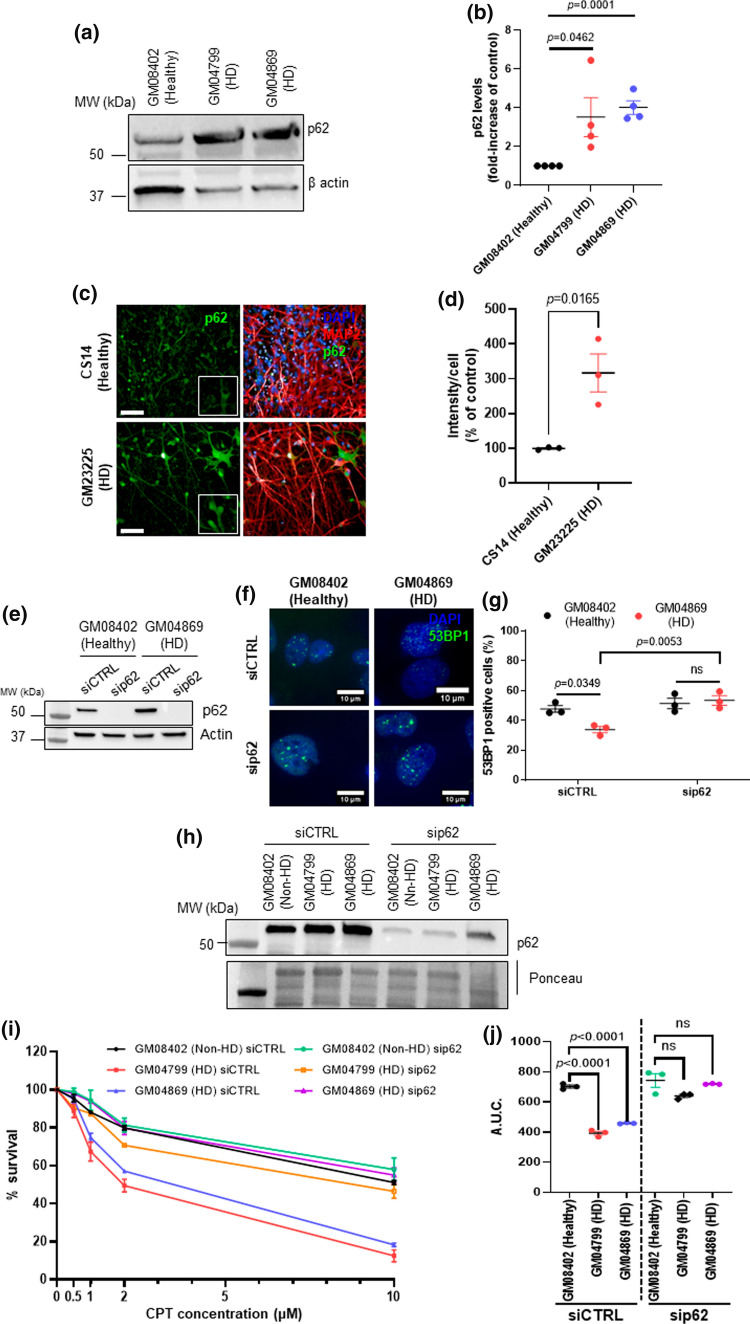
P62 depletion restores 53BP1 signaling and ameliorates hypersensitivity to TOP1 DNA damage. **a** Whole cell extracts from healthy (GM08402) and HD patient fibroblasts (GM04799, 42 CAG and GM04869, 47 CAG) were analyzed by western blotting using a p62 antibody. Actin was used as loading control. **b** Fold-change of p62 levels relative to healthy cells was calculated and analyzed by Student’s *t*-test. Error bars: ± s.e.m, *n* = 4. **c** Immunofluorescence images of healthy and HD striatal neurons stained for p62. DAPI shows nuclear staining and MAP2 represents a neuronal marker (scale bar: 10 μm). **d** Intensity of p62 per cell was quantified and shown as % of healthy. Data were analyzed by Student’s *t*-test (error bars: ± s.e.m from three technical repeats). **e** Healthy (GM08402) and HD fibroblasts (GM04869) were transfected with control siRNA particles (siCTRL) or targeting p62 (sip62) and analyzed by western blotting using a p62 antibody. Actin staining shows loading. **f** 53BP1-immunofluorescence images of healthy and HD fibroblasts transfected with sip62 or siCTRL after treatment with 0.5 μM of CPT for 1 h (scale bar: 10 μm). **g** The percentage of 53BP1 positive cells (> 5 foci) was quantified and analyzed by One-way ANOVA, followed by post hoc Tukey’s multiple comparisons test (n = 3, ± s.e.m.). **h** Western blotting analysis of healthy (GM08402) and HD fibroblasts (HD1: GM04799 and HD2: GM04869) after siRNA transfection, using an anti-p62 antibody. Ponceau indicates loading. **i** Sensitivity to CPT of healthy and HD patient fibroblasts transfected with either siCTRL or sip62 was monitored by CellTiter-Blue® assay after 96 h treatment CPT (0–10 µM). *Y*-axis: mean percentage survival (% survival) of *n* = 3 plotted against CPT concentration. % survival was assessed by normalizing against the corresponding untreated condition (0 µM). **j** The area under the curve (A.U.C.) was calculated for each condition and plotted as average as in [25]. The data were analyzed by Student’s *t*-test (error bars: ± s.e.m; *n* = 3). *ns* nonsignificant

Defects in selective autophagy is a common feature of neurodegenerative diseases [15, 22, 61, 62]. p62 is an autophagy receptor that accumulates in autophagy-defective cells [63]. A previous study demonstrated that p62 accumulation suppressed DNA damage-induced ubiquitination of H2A in autophagy deficient cells [39]. We therefore examined the levels of p62 in our HD models. Western blotting analysis of HD patient-derived skin fibroblasts indicates an increased level of p62 in both HD cells in comparison with the healthy cells (Fig. 6a, b). Similarly, analysis of the p62 levels in GABAergic neurons by immunofluorescence also showed accrued p62 levels in HD neurons (Fig. 6c, d). Since 53BP1 recruitment to the chromatin depends on H2A ubiquitination we aimed to further elucidate if the attenuated 53BP1 recruitment in HD cells after DNA damage is related to p62. We depleted p62 using small interfering RNA (siRNA) in healthy and in GM04869 HD fibroblasts (Fig. 6e). After depletion of p62, 53BP1 levels of HD cells were comparable to those of healthy fibroblasts, further confirming the negative effects of p62 in 53BP1 signaling in HD cells (Fig. 6f, g). We then assessed the effects of p62 depletion in cell viability (Fig. 6j). As in Fig. 3e, HD patient derived fibroblasts exhibited a reduction in survival in comparison with healthy fibroblasts following CPT treatment. However, depletion of p62 restored cell viability in both HD cells (Fig. 6i, j).

### Pharmacologic disruption of p62:RNF168 binding rescues 53BP1 signaling in HD cellular models

**Fig. 7:**
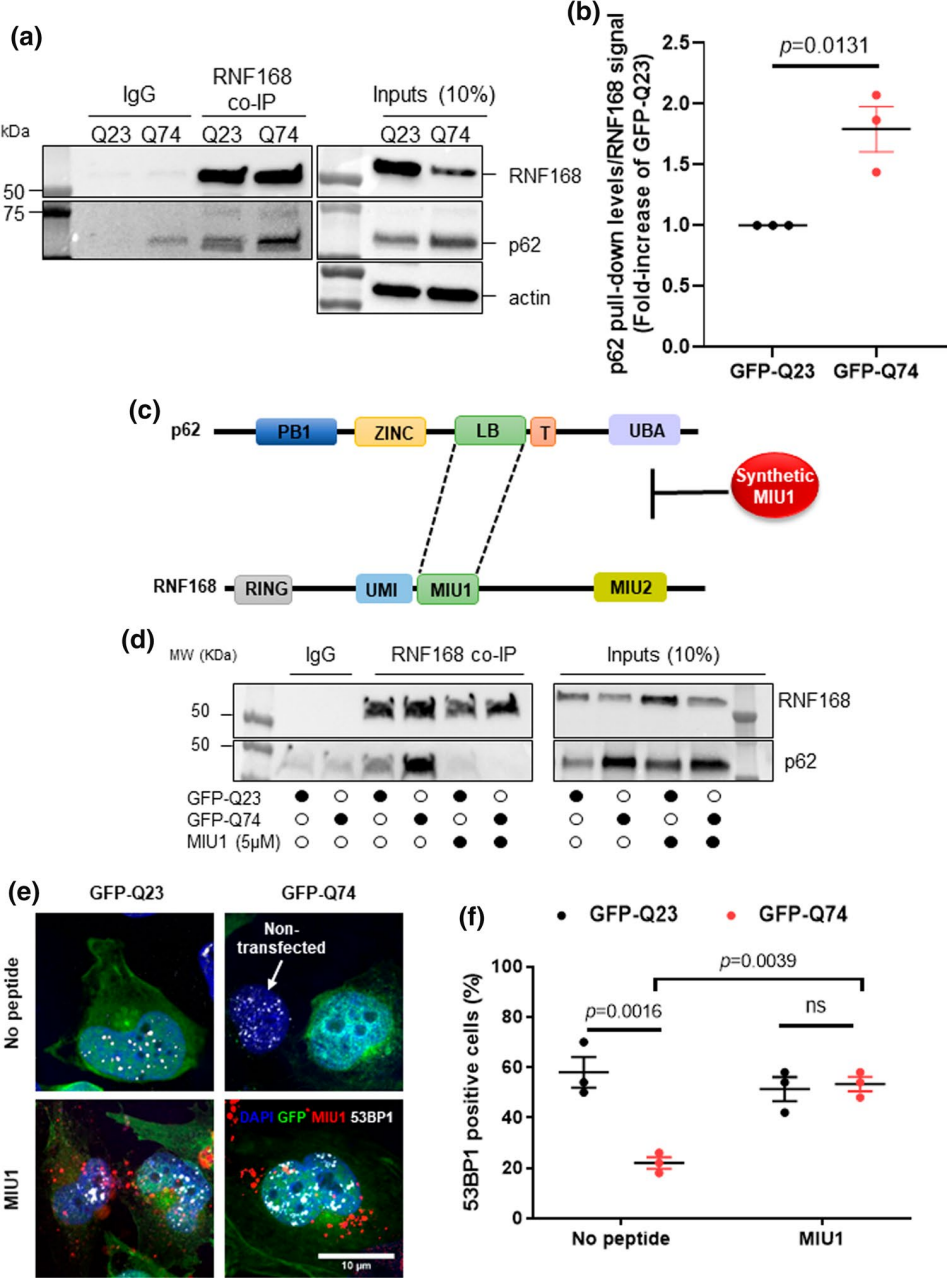
Pharmacological disruption of p62:RNF 168 binding rescues 53BP1 recruitment in response to DNA damage in cells expressing mutant huntingtin. **a** RNF168 co-immunoprecipitation (RNF168 co-IP) of nuclear fractions of HEK293 co-transfected with GFP-Q23 or GFP-74. Left: Western blotting shows interaction between RNF168 and p62. Right: 10% of lysates were analyzed by western blotting after incubation with anti-RNF168 and anti-p62 antibodies. Actin shows loading control. (Q23: wild-type huntingtin. Q74: mutant huntingtin). **b** p62 pull-down levels were quantified and normalized to the amount of p62 present in the inputs. This value was normalized against the bait RNF168 levels. The data are shown as fold-change of GFP-Q23. The data were analyzed by Student’s *t*-test and shown as average ± s.e.m.; *n* = 3. **c** Schematic representation of p62 and RNF168 interaction. LB domain of p62 interacts with MIU1 of RNF168. The interaction between p62 and RNF168 is interrupted by a recombinant rhodamine-tagged peptide that mimics MIU1 domain of RNF168. **d** RNF168 Co-IP was performed in nuclear fractions of HEK293 transfected with GFP-Q23 or GFP-74. Left: Western blot shows the interaction between RNF168 and p62 (lanes 3 and 4). The interaction is perturbed after incubation with 5 μM MIU1 peptide for 24 h (lanes 5 and 6). Right: 10% of lysates were analyzed by western blotting with anti-RNF168 and anti-p62 antibodies. (Q23: wild-type huntingtin. Q74: mutant huntingtin). **e** MRC5 cells were transfected with GFP-Q23 or GFP-Q74 plasmids and exposed to 5 µM mock (DMSO)/ rhodamine-tagged MIU1. Cells were treated with 10 μM CPT for 1 h and analyzed by immunofluorescence (scale bar: 10 µm). **f** The percentage 53BP1 positive cells (> 5 foci) were quantified and analyzed by One-way ANOVA, followed by Tukey’s multiple comparisons test. Data are shown as average ± s.e.m; *n* = 3 (50 GFP-expressing cells/replicate). *p < 0.05; **p < 0.01; *ns* nonsignificant

Since p62 accumulation has been reported to bind to RNF168 [22, 39], which may explain the reduced H2A ubiquitination and consequent defective 53BP1 recruitment, we next examined whether mutant HTT expression increases p62 binding to RNF168. To do this, we immobilized RNF168 specific antibody to magnetic beads and incubated with nuclear extracts of HEK293 cells expressing either GFP-Q23 or GFP-Q74. As before, we used HEK293 cells due to the ease of transfection and ability to generate sufficient cell lysate. Western blotting analysis of the eluates showed that p62 strongly co-immunoprecipitated with RNF168 in nuclear extracts from cells expressing mutant HTT, whereas cells expressing wild-type HTT showed less p62 pulled-down by RNF168 (Fig. 7a, b). These results suggest that mutant HTT expression promotes p62 interaction with RNF168. Therefore, the defective 53BP1 response to CPT observed might be explained by reduced RNF168 activity due to increased p62:RNF168 interaction promoted by mutant HTT. Interestingly, we also observed reduced RNF168 levels in cells overexpressing mutant HTT (inputs blot). Although the expression levels of RNF168 were decreased in Q74 cells, the amount of bait-RNF168 bound to the beads was similar in both Q23 and Q74 samples, indicating that comparable levels of RNF168 pulled-down different amounts of p62.

The LIN-11, Isl1 and MEC-3 (LIM) protein-binding (LB) domain of p62 interacts with the MIU motif 1 (MIU1) of RNF168 [39]. In order to test if the disruption of p62 binding to RNF168 rescues 53BP1 signaling in cells expressing mutant HTT we designed a rhodamine-tagged synthetic MIU1 peptide which mimics the MIU1 domain of RNF168 (Fig. 7c). Co-immunoprecipitation assays were performed to test whether the synthetic MIU1 competes against RNF168 and prevents binding of p62. HEK293 cells were transfected with GFP-Q23 or GFP-Q74 plasmids and treated with either synthetic MIU1 or DMSO (Mock) for 24 h. Consistent with previous data, increased levels of p62 were pulled down by RN168 in nuclear extracts from cells expressing GFP-Q74 comapred to GFP-Q23 cells. Notably, treatment with MIU1 was able to suppress the RNF168:p62 interaction in both mutant and wild-type HTT cells (Fig. 7d). We then examined whether disrupting p62 binding to RNF168 with synthetic MIU1 rescues the defective 53BP1 phenotype in HD models. Cells expressing either GFP-Q23 or GFP-Q74 were exposed to 5 µM recombinant MIU1 peptide for 24 h and then treated with 10 µM CPT for 1 h. In cells expressing GFP-Q23, 53BP1 foci formed normally after CPT treatment in both mock and MIU1 treated cells. In contrast, mock-treated GFP-Q74 cells exhibited less 53BP1 foci in response to CPT. After exposure to MIU1 peptide, cells expressing GFP-Q74 re-established 53BP1 response to CPT treatment (Fig. 7e, f). These results suggest that in HD, RNF168 activity is reduced due to increased p62 binding, leading to defects in 53BP1 recruitment during DNA repair signaling. Together, we conclude that treatment with a synthetic peptide that mimics the RNF168 binding site to p62 was able to liberate RNF168 and rescue defective 53BP1 signaling in HD models.

## Discussion

In this study, we first identified that mutant HTT caused deficient 53BP1 recruitment to the nucleus of cells after TOP1-induced DNA breaks in different HD cellular models, including striatal neurons from HD patients. Simultaneously, we have observed that HD fibroblasts seem to repair the damage induced by CPT at a slower rate than healthy cells, as suggested by the increased percentage of γH2AX positive cells at later recovery time points and the increased neutral comet tail moment. These results indicate HD cells struggle to repair TOP1cc-induced DNA damage and accumulate TOP1cc which are further turned into cytotoxic DSBs. Both patient-derived HD fibroblasts and HD GABAergic neurons exhibited augmented activation of apoptotic markers in response to CPT treatment, indicating HD cells are hypersensitive to CPT, which triggers apoptotic cell death. Together, our observations suggest that in HD cells, the DDR signaling triggered by TOP1-related DSBs is compromised, contributing to increased cell death. Notably, TOP1-mediated DNA lesions are physiologically relevant since TOP1cc-induced DNA breaks are endogenous and common threats to neuronal genome stability [21–23, 48].

Our findings are consistent with other studies showing disruption of DSB repair cascade by mutant HTT. An early study demonstrated that oxidative damage induced exaggerated activation of ATM signaling, concomitant with the increased presence of γH2AX and the DNA lesions [64]. Another study described a mechanism by which mutant HTT sequesters Ku70, a component of the NHEJ machinery [30]. Ku70, in complex with Ku80 is responsible for the downstream recruitment of DNA-PK. Binding of mutant HTT prevented Ku70-Ku80 and Ku70-DNA interactions, thus impairing DNA-PK activity [30]. In addition, inclusion bodies formed by aggregated HTT were shown to attract 53BP1, which also contributes for deficient 53BP1 recruitment to the damaged sites [29]. A study conducted by the Foray group showed HD fibroblasts yield fewer 53BP1 foci in comparison with healthy fibroblasts after irradiation, indicating a deficient recruitment of 53BP1 to DSBs, which is consistent with our findings [35]. Faulty 53BP1 signaling in HD fibroblasts was found to be associated with the lack of pATM signaling after irradiation, which was suggested to be caused by mutant HTT-mediated sequestration of ATM to the cytoplasm [35]. Similarly, defective pATM foci formation was observed in both RNA repeat expansion (RRE) and dipeptide repeats (DPR) models of *C9orf72* ALS/FTD [22]. A recent study demonstrated that poly-glycine–alanine (poly-GA), DPRs frequently observed in *C9orf72* ALS/FTD patients, induced cytoplasmic sequestration of pATM, which is again consistent with our observations, suggesting that similar mechanisms might play a role in promoting neurodegeneration in different disorders [65]. Activation of ATM is an upstream event in the DSB repair cascade [66]. Sequestration of pATM might in part explain the reduced 53BP1 response in our models since ATM-mediated phosphorylation events promotes 53BP1 recruitment and, consequently, NHEJ repair [67–69]. However, evidence suggests 53BP1 occupancy at DNA damage sites is also necessary for the retention of ATM at the chromatin and to propagate ATM-dependent signaling [46, 70]. Together with these studies, our results suggest the presence of mutant HTT interferes with several steps of the ATM-mediated DSB repair pathway, which potentially impact the repair of TOP1-induced DNA lesions in HD brains, given the importance of ATM in preventing TOP1-mediated neuronal genomic instability [21, 71]. Additional to the canonical role of ATM in DSB signaling, ATM is also necessary for the degradation of DNA-trapped TOP1 prior to TDP1-mediated excision, in a kinase independent way. As such, absence of ATM results in accumulation of TOP1cc [21]. Consistent with defective ATM signaling, we showed HD cells accumulate endogenous and CPT-driven TOP1cc. Whether this results from mtHTT-mediated hinderance of ATM functions is unclear.

We also demonstrated that both ectopic expression of mutant HTT and HD patient fibroblasts exhibited attenuated H2A ubiquitination at baseline. However, global H2A ubiquitination was unchanged after CPT treatment. Further analysis demonstrated that cells expressing mutant HTT displayed weak H2AK13/K15 ubiquitination after CPT treatment, supporting our hypothesis that impaired 53BP1 recruitment is caused by defects in ubiquitination of specific lysine residues. The recruitment of DDR factors is highly dependent on chromatin changes. After DNA damage, a series of histone modifications occur to regulate and facilitate DNA repair [72]. RNF168-mediated H2A ubiquitination on K15 promotes the recruitment 53BP1 to the damage sites [45, 54]. Given that, it is expected that defects in RNF168-mediated H2A ubiquitination impacts the subsequent 53BP1 recruitment to the damage sites. An example is shown in radiosensitivity, immunodeficiency, dysmorphic facial features, and learning difficulties (RIDDLE). syndrome, which is associated with abortive RNF168 activity [73]. Cells from RIDDLE patients display defects in 53BP1 signaling concomitant with absent RNF168-mediated H2A ubiquitination [73–75]. Our findings are reminiscent to the defects in 53BP1 signaling due to lack of RN168 activity observed in RIDDLE syndrome [73, 74, 76]. Patients with RIDDLE syndrome clinically manifest learning impairment and ataxia, the latter mimicking an A-T phenotype [76]. This demonstrates a neuroprotective role of RNF168, where its deficient activity leads to neurological phenotypes. Moreover, the fact that both RIDDLE, and HD cells are characterized by increased radiosensitivity further suggests RNF168 contribution to the pathogenesis of HD [35, 73].

Since p62 accumulation was shown to prevent H2A ubiquitination, we next examined p62 levels in our models [39]. We have noticed a possible predisposition for p62 accumulation in fibroblasts and striatal neurons from HD patients. In agreement with our findings, increased p62 levels have been reported in several HD models, including in mouse Neuro2a cells expressing 150Q [77] and in striatal neurons of HdhQ200 mice [78]. A recent report has also described increased p62 expression in both HEK293 cells transduced with mutant HTT (66Q) and in striatal neurons expressing mutant HTT [79]. Another study in R6/1 HD mice models, has shown reduced p62 levels in the mice brain in the early stages of the disease [80]. In the later stages, however, p62 accumulation was detected in the nuclei of striatal neurons, suggesting an age-dependent accumulation of p62 as the disease progresses, which is modulated by p62 aberrant interaction with mutant HTT [80]. Next, we showed that p62 knockdown using siRNA ameliorated the adverse effects of p62 in HD fibroblasts, as it reinstalled 53BP1 foci and cell viability upon CPT treatment. These results are in agreement with our recent report showing that in *C9orf72*-ALS cells, depletion of p62 restores ATM signaling and NHEJ repair as it re-establishes RNF168-mediated H2A ubiquitination and 53BP1 recruitment [22]. Depletion of p62 also seemed to improve the survival of HD fibroblasts in response to DNA damage. Notably, our findings are reminiscent of a previous study showing that ablation of p62 reduced nuclear inclusions in the striatum and extended the life span of HD mice [81]. The reduced nuclear polyQ inclusions and increased cytoplasmic aggregates followed by p62 depletion, further indicates p62 is involved in the autophagic clearance of cytosolic polyQ inclusions [81]. Defects in clearing toxic aggregates is a risk factor for neurodegenerative diseases such as HD [37]. Therefore, although decreasing p62 levels restored 53BP1 response and led to better survival in HD cells after DNA damage, the possible negative impact of p62 depletion requires further investigation.

**Fig. 8:**
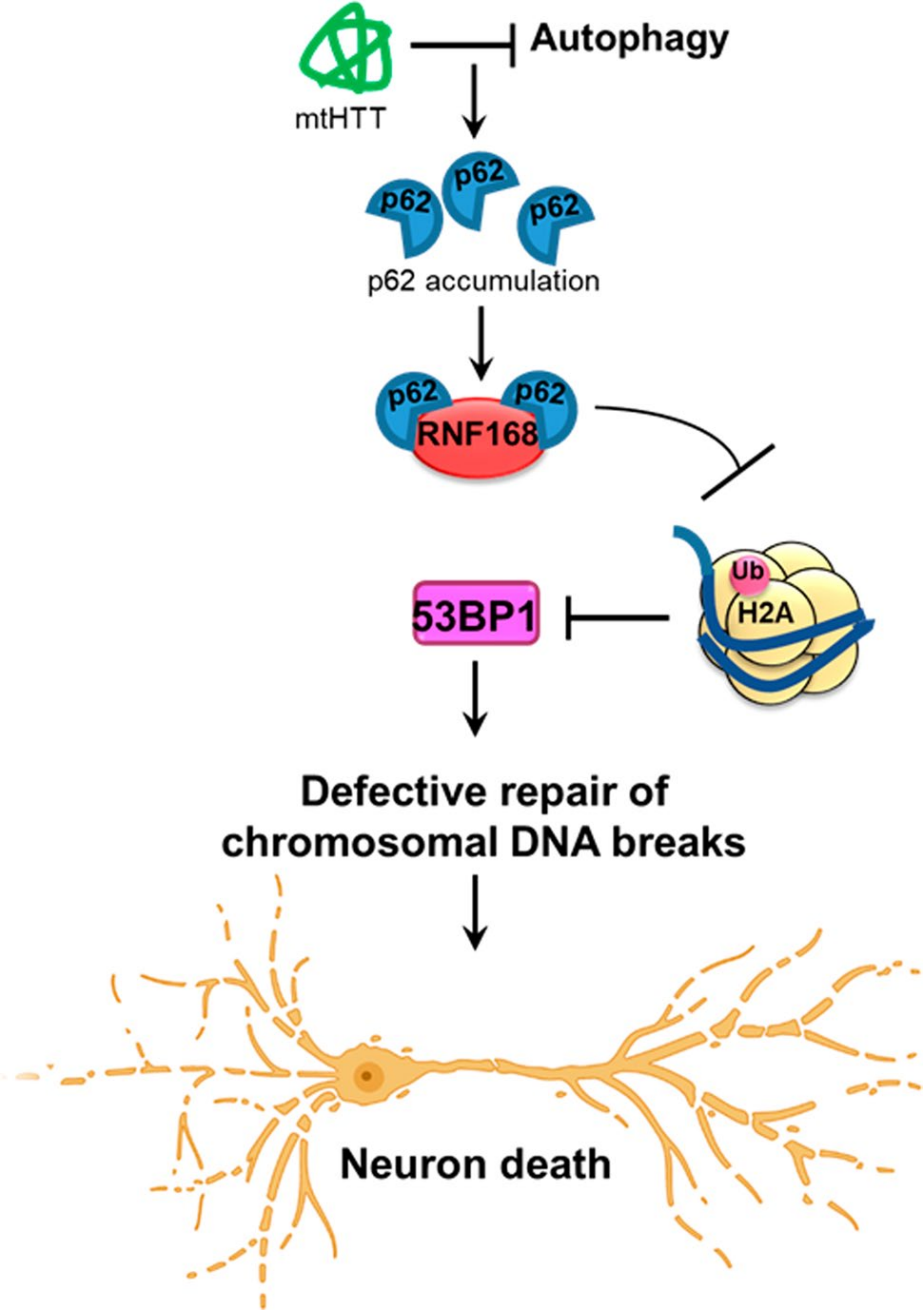
Proposed model showing defective repair of chromosomal DNA breaks in Huntington’s disease. Mutant huntingtin protein (mtHTT) causes defects in autophagy, promoting accumulation of the cargo receptor protein, p62. Increased p62 levels then proceed to inactivate the E3 ubiquitin ligase activity of RNF168 during the repair of chromosomal DNA breaks. This leads to insufficient histone H2AK13/K15 ubiquitination which is necessary for the recruitment of repair factors such as 53BP1. By failing to recruit 53BP1, cells are left with unrepaired damage which lead to premature neuronal death

Finally, we showed that ectopic expression of mutant HTT promoted p62 interaction with RNF168. A link between defective RNF168 activity and faulty autophagy mechanisms has been described, where accumulation of nuclear p62 due to loss of autophagy mechanisms negatively impacts chromatin ubiquitination during DDR, as RNF168 activity is disrupted by p62 direct interaction [39]. Nonetheless, it remains unclear what the link is between CAG expansions in mutant HTT and the increased p62:RNF168 interaction. Mutant HTT directly interferes with autophagic degradation of cytosolic cargo by preventing autophagolysosomal-mediated cargo recognition [61, 82]. Furthermore, a previous study has demonstrated that aggregation of mutant HTT inhibits autophagy, which is accompanied by p62 accumulation [79]. Another report shows that inhibition of autophagy promotes translocation of p62 the nucleus, and its depletion restored RNF168-induced H2A ubiquitination, which further agrees with our findings [83]. Hence, we speculate mutant HTT causes defects in autophagy mechanisms, causing a build-up of p62, which interferes with RNF168 activity and consequently leads to defective H2A ubiquitination and defective DNA repair (Fig. 8). Others have shown that in quiescent cells, induction of co-transcriptional DSBs by CPT treatment promotes DNA-PK-mediated H2A(X) ubiquitination. Inhibition of DNA-PK prevented H2A ubiquitination and reduced pATM recruitment [71]. Given our findings, it would be interesting to investigate the role of DNA-PK in H2A ubiquitination in the context of HD.

Remarkably, disruption of RNF168 interaction with p62 by using a synthetic peptide that mimics RNF168 MIU1 domain successfully reinstated 53BP1 response to TOP1-induced DNA damage in cells expressing mutant HTT, further supporting that p62 negatively regulates RNF168 activity and the resultant recruitment of downstream DDR components. Although recombinant MIU1 also disrupted RNF168:p62 interaction in cells overexpressing wild-type HTT, no differences were observed in 53BP1 signaling. Our data and the study conducted by the Zhao group indicate that the interaction between RNF168 and p62 also happens under physiological levels [39]. However, the physiological role of this interaction, particularly in the context of DNA repair remains elusive. Even so, our results suggest disruption of RNF168:p62 interaction might constitute a new therapeutic strategy for HD. Nonetheless, further examination is necessary to assess possible toxic effects of this approach. The use of small peptides to inhibit aberrant protein:protein interactions (PPIs) is a promising approach in neurodegenerative disorders [84]. However, finding small molecules that are stable, cell/blood–brain barrier permeable, and able to modulate PPIs is a challenging task. A recent study described an abnormal interaction between mutant SOD1 and Derlin-1 was responsible for causing motor neuron cell death in ALS models [85]. By taking advantage of the time resolved fluorescence resonance energy transfer (TR-FRET) technology, the authors developed a high-throughput system to screen the efficacy of 160,000 compounds in inhibiting mutant SOD1:Derlin-1 interaction. Excitingly, the screening identified one compound analog that was able to prevent the aberrant interaction between mutant SOD1 and Derlin-1 and showed good permeability and metabolic stability. Moreover, treatment with this analog prevented motor neuron death in ALS models [85]. Similar strategies could be implemented to screen for potential small molecules that inhibit of RNF168:p62 interaction and test their therapeutic effects in HD models.

In conclusion, we unveiled a crosstalk between autophagy defects and faulty DDR as a consequence of polyQ repeat expansions in HD. Our work suggests that similar molecular mechanisms contribute to neurodegeneration by increasing genomic instability in both HD and *C9orf72*-ALS/FTD, thus indicating that autophagy-mediated DDR defects might be a common pathological phenotype of polynucleotide expansion neurodegenerative disorders.

### Supplementary Information

Below is the link to the electronic supplementary material.

Supplementary file1 (DOCX 4185 KB)

## Data Availability

Enquiries about data availability should be directed to the authors.

## References

[1] Bates GP, Dorsey R, Gusella JF (2015). Huntington disease. Nat Rev Dis Prim.

[2] Tabrizi SJ, Ghosh R, Leavitt BR (2019). Huntingtin lowering strategies for disease modification in Huntington’s disease. Neuron.

[3] Lopes C, Aubert S, Bourgois-Rocha F (2016). Dominant-negative effects of adult-onset huntingtin mutations alter the division of human embryonic stem cells-derived neural cells. PLoS ONE.

[4] Suberbielle E, Sanchez PE, Kravitz AV (2013). Physiologic brain activity causes DNA double-strand breaks in neurons, with exacerbation by amyloid-β. Nat Neurosci.

[5] Massey TH, Jones L (2018) The central role of DNA damage and repair in CAG repeat diseases. Dis Model Mech 11:dmm031930. 10.1242/dmm.031930 10.1242/dmm.031930 PMC5818082 29419417

[6] Madabhushi R, Gao F, Pfenning AR (2015). Activity-induced DNA breaks govern the expression of neuronal early-response genes. Cell.

[7] Daroui P, Desai SD, Li TK (2004). Hydrogen peroxide induces topoisomerase I-mediated DNA damage and cell death. J Biol Chem.

[8] García-Muse T, Aguilera A (2019). R loops: from physiological to pathological roles. Cell.

[9] McKinnon PJ (2016). Topoisomerases and the regulation of neural function. Nat Rev Neurosci.

[10] Zaksauskaite R, Thomas RC, van Eeden F, El-Khamisy SF (2021) Tdp1 protects from topoisomerase 1–mediated chromosomal breaks in adult zebrafish but is dispensable during larval development. Sci Adv 7:eabc4165. 10.1126/sciadv.abc4165 10.1126/sciadv.abc4165 PMC7846158 33514542

[11] Alagoz M, Chiang S, Sharma A, El-khamisy SF (2013). ATM deficiency results in accumulation of DNA-topoisomerase I covalent intermediates in neural cells. PLoS ONE.

[12] Cristini A, Ricci G, Britton S (2019). Dual Processing of R-loops and topoisomerase I induces transcription-dependent DNA double-strand breaks. Cell Rep.

[13] Ashour ME, Allam W, Elsayed W (2021). High temperature drives topoisomerase mediated chromosomal break repair pathway choice. Cancers (Basel).

[14] McKinnon PJ (2009). DNA repair deficiency and neurological disease. Nat Rev Neurosci.

[15] Abugable AA, Morris JLM, Palminha NM (2019). DNA repair and neurological disease: from molecular understanding to the development of diagnostics and model organisms. DNA Repair (Amst).

[16] Biton S, Gropp M, Itsykson P (2007). ATM-mediated response to DNA double strand breaks in human neurons derived from stem cells. DNA Repair (Amst).

[17] Shiloh Y, Ziv Y (2013). The ATM protein kinase: regulating the cellular response to genotoxic stress, and more. Nat Rev Mol Cell Biol.

[18] Sun Y, Curle AJ, Haider AM, Balmus G (2020). The role of DNA damage response in amyotrophic lateral sclerosis. Essays Biochem.

[19] Jackson SP, Bartek J (2009). The DNA-damage response in human biology and disease. Nature.

[20] Kok JR, Palminha NM, Dos Santos SC (2021). DNA damage as a mechanism of neurodegeneration in ALS and a contributor to astrocyte toxicity. Cell Mol Life Sci.

[21] Katyal S, Lee Y, Nitiss KC (2014). Aberrant topoisomerase-1 DNA lesions are pathogenic in neurodegenerative genome instability syndromes. Nat Neurosci.

[22] Walker C, Herranz-Martin S, Karyka E (2017). C9orf72 expansion disrupts ATM-mediated chromosomal break repair. Nat Neurosci.

[23] El-Khamisy SF, Saifi GM, Weinfeld M, Caldecott KW (2005). Defective DNA single-strand break repair in spinocerebellar ataxia with axonal neuropathy-1. Nature.

[24] Pommier Y (2006). Topoisomerase I inhibitors: camptothecins and beyond. Nat Rev Cancer.

[25] Balmus G, Pilger D, Coates J (2019). ATM orchestrates the DNA-damage response to counter toxic non-homologous end-joining at broken replication forks. Nat Commun.

[26] Sordet O, Redon CE, Guirouilh-Barbat J (2009). Ataxia telangiectasia mutated activation by transcription- and topoisomerase I-induced DNA double-strand breaks. EMBO Rep.

[27] Das BB, Antony S, Gupta S (2009). Optimal function of the DNA repair enzyme TDP1 requires its phosphorylation by ATM and/or DNA-PK. EMBO J.

[28] Jeon GS, Kim KY, Hwang YJ (2012). Deregulation of BRCA1 leads to impaired spatiotemporal dynamics of γ-H2AX and DNA damage responses in Huntington’s disease. Mol Neurobiol.

[29] Ben YA, Risheq M, Novoplansky O (2017). Ubiquitin accumulation on disease associated protein aggregates is correlated with nuclear ubiquitin depletion, histone De-ubiquitination and impaired DNA damage response. PLoS ONE.

[30] Enokido Y, Tamura T, Ito H (2010). Mutant huntingtin impairs Ku70-mediated DNA repair. J Cell Biol.

[31] Maiuri T, Suart CE, Hung CLK (2019). DNA damage repair in Huntington’s disease and other neurodegenerative diseases. Neurotherapeutics.

[32] GeM-HD GM of HDC (2015). Identification of genetic factors that modify clinical onset of Huntington’s disease. Cell.

[33] Lu XH, Mattis VB, Wang N, et al (2014) Targeting ATM ameliorates mutant Huntingtin toxicity in cell and animal models of Huntington’s disease. Sci Transl Med 6:268ra178. 10.1126/scitranslmed.3010523 10.1126/scitranslmed.3010523 25540325

[34] Victor MB, Richner M, Olsen HE (2018). Striatal neurons directly converted from Huntington’s disease patient fibroblasts recapitulate age-associated disease phenotypes. Nat Neurosci.

[35] Ferlazzo MLL, Sonzogni L, Granzotto A (2014). Mutations of the Huntington’s disease protein impact on the ATM-dependent signaling and repair pathways of the radiation-induced DNA double-strand breaks: corrective effect of statins and bisphosphonates. Mol Neurobiol.

[36] McKinnon PJ (2017). Genome integrity and disease prevention in the nervous system. Genes Dev.

[37] Martin DDO, Ladha S, Ehrnhoefer DE, Hayden MR (2015). Autophagy in Huntington disease and huntingtin in autophagy. Trends Neurosci.

[38] Lee H, Kim MN, Ryu KY (2017). Effect of p62/SQSTM1 polyubiquitination on its autophagic adaptor function and cellular survival under oxidative stress induced by arsenite. Biochem Biophys Res Commun.

[39] Wang Y, Zhang N, Zhang L (2016). Autophagy regulates chromatin ubiquitination in DNA damage response through elimination of SQSTM1/p62. Mol Cell.

[40] Du ZW, Chen H, Liu H (2015). Generation and expansion of highly pure motor neuron progenitors from human pluripotent stem cells. Nat Commun.

[41] Mangiarini L, Sathasivam K, Seller M (1996). Exon 1 of the HD gene with an expanded cAG repeat is sufficient to cause a progressive neurological phenotype in transgenic mice. Cell.

[42] Li X, Standley C, Sapp E (2009). Mutant Huntingtin impairs vesicle formation from recycling endosomes by interfering with Rab11 activity. Mol Cell Biol.

[43] Marchina E, Misasi S, Bozzato A (2014). Gene expression profile in fibroblasts of Huntington’s disease patients and controls. J Neurol Sci.

[44] Hu J, Liu J, Yu D (2014). Exploring the effect of sequence length and composition on allele-selective inhibition of human huntingtin expression by single-stranded silencing RNAs. Nucleic Acid Ther.

[45] Fradet-Turcotte A, Canny MD, Escribano-Díaz C (2013). 53BP1 is a reader of the DNA-damage-induced H2A Lys 15 ubiquitin mark. Nature.

[46] Baldock RA, Day M, Wilkinson OJ (2015). ATM localization and heterochromatin repair depend on direct interaction of the 53BP1-BRCT2 domain with γH2AX. Cell Rep.

[47] Maiuri T, Mocle AJ, Hung CL (2017). Huntingtin is a scaffolding protein in the ATM oxidative DNA damage response complex. Hum Mol Genet.

[48] Carlessi L, Poli EF, Bechi G (2014). Functional and molecular defects of hiPSC-derived neurons from patients with ATM deficiency. Cell Death Dis.

[49] Pommier Y, Redon C, Rao VA (2003). Repair of and checkpoint response to topoisomerase I-mediated DNA damage. Mutat Res Fundam Mol Mech Mutagen.

[50] Jimenez-Sanchez M, Licitra F, Underwood BR, Rubinsztein DC (2017). Huntington’s disease: mechanisms of pathogenesis and therapeutic strategies. Cold Spring Harb Perspect Med.

[51] Ehrlich ME (2012). Huntington’s disease and the striatal medium spiny neuron: cell-autonomous and non-cell-autonomous mechanisms of disease. Neurotherapeutics.

[52] Zheng P, Kozloski J (2017). Striatal network models of Huntington’s disease dysfunction phenotypes. Front Comput Neurosci.

[53] Lin L, Yuan J, Sander B, Golas MM (2015). In vitro differentiation of human neural progenitor cells into striatal GABAergic neurons. Stem Cells Transl Med.

[54] Hu Y, Wang C, Huang K (2014). Regulation of 53BP1 protein stability by RNF8 and RNF168 is important for efficient DNA double-strand break repair. PLoS ONE.

[55] Hu Q, Botuyan MV, Cui G (2017). Mechanisms of ubiquitin-nucleosome recognition and regulation of 53BP1 chromatin recruitment by RNF168/169 and RAD18. Mol Cell.

[56] Kashiwagi H, Shiraishi K, Sakaguchi K (2018). Repair kinetics of DNA double-strand breaks and incidence of apoptosis in mouse neural stem/progenitor cells and their differentiated neurons exposed to ionizing radiation. J Radiat Res.

[57] Zimmermann M, De Lange T (2014). 53BP1: Pro choice in DNA repair. Trends Cell Biol.

[58] Walser F, Mulder MPC, Bragantini B (2020). Ubiquitin phosphorylation at Thr12 modulates the DNA damage response. Mol Cell.

[59] Uckelmann M, Sixma TK (2017). Histone ubiquitination in the DNA damage response. DNA Repair (Amst).

[60] Pierzynowska K, Gaffke L, Hać A (2018). Correction of Huntington’s disease phenotype by genistein-induced autophagy in the cellular model. Neuromol Med.

[61] Martinez-Vicente M, Talloczy Z, Wong E (2010). Cargo recognition failure is responsible for inefficient autophagy in Huntington’s disease. Nat Neurosci.

[62] Bhat KP, Yan S, Wang C-E (2014). Differential ubiquitination and degradation of huntingtin fragments modulated by ubiquitin-protein ligase E3A. Proc Natl Acad Sci.

[63] Lee YJ, Chou TF, Pittman SK (2017). Keap1/Cullin3 modulates p62/SQSTM1 activity via UBA domain ubiquitination. Cell Rep.

[64] Giuliano P (2003). DNA damage induced by polyglutamine-expanded proteins. Hum Mol Genet.

[65] Nihei Y, Mori K, Werner G (2019). Poly-glycine–alanine exacerbates C9orf72 repeat expansion-mediated DNA damage via sequestration of phosphorylated ATM and loss of nuclear hnRNPA3. Acta Neuropathol.

[66] Blackford AN, Jackson SP (2017). ATM, ATR, and DNA-PK: the trinity at the heart of the DNA damage response. Mol Cell.

[67] Feng L, Li N, Li Y (2015). Cell cycle-dependent inhibition of 53BP1 signaling by BRCA1. Cell Discov.

[68] Panier S, Boulton SJ (2014). Double-strand break repair: 53BP1 comes into focus. Nat Rev Mol Cell Biol.

[69] Mailand N, Bekker-Jensen S, Faustrup H (2007). RNF8 ubiquitylates histones at DNA double-strand breaks and promotes assembly of repair proteins. Cell.

[70] Lee JH, Goodarzi AA, Jeggo PA, Paull TT (2010). 53BP1 promotes ATM activity through direct interactions with the MRN complex. EMBO J.

[71] Cristini A, Park J-H, Capranico G (2016). DNA-PK triggers histone ubiquitination and signaling in response to DNA double-strand breaks produced during the repair of transcription-blocking topoisomerase I lesions. Nucleic Acids Res.

[72] Thorslund T, Ripplinger A, Hoffmann S (2015). Histone H1 couples initiation and amplification of ubiquitin signalling after DNA damage. Nature.

[73] Stewart GS, Panier S, Townsend K (2009). The RIDDLE syndrome protein mediates a ubiquitin-dependent signaling cascade at sites of DNA damage. Cell.

[74] Stewart GS, Stankovic T, Byrd PJ (2007). RIDDLE immunodeficiency syndrome is linked to defects in 53BP1-mediated DNA damage signaling. Proc Natl Acad Sci USA.

[75] Pietrucha B, Heropolitanska-Pliszka E, Geffers R (2017). Clinical and biological manifestation of RNF168 deficiency in two polish siblings. Front Immunol.

[76] Devgan SS, Sanal O, Doil C (2011). Homozygous deficiency of ubiquitin-ligase ring-finger protein RNF168 mimics the radiosensitivity syndrome of ataxia-telangiectasia. Cell Death Differ.

[77] Nagaoka U, Kim K, Jana NR (2004). Increased expression of p62 in expanded polyglutamine-expressing cells and its association with polyglutamine inclusions. J Neurochem.

[78] Heng MY, Detloff PJ, Paulson HL, Albin RL (2010). Early alterations of autophagy in Huntington disease-like mice. Autophagy.

[79] Pircs K, Petri R, Madsen S (2018). Huntingtin aggregation impairs autophagy, leading to argonaute-2 accumulation and global microRNA dysregulation. Cell Rep.

[80] Rué L, López-soop G, Gelpi E, Martínez-vicente M (2013). Neurobiology of Disease Brain region- and age-dependent dysregulation of p62 and NBR1 in a mouse model of Huntington ’ s disease. Neurobiol Dis.

[81] Kurosawa M, Matsumoto G, Kino Y (2015). Depletion of p62 reduces nuclear inclusions and paradoxically ameliorates disease phenotypes in Huntington’s model mice. Hum Mol Genet.

[82] Rui YN, Xu Z, Patel B (2015). Huntingtin functions as a scaffold for selective macroautophagy. Nat Cell Biol.

[83] Wang L, Howell MEA, Sparks-Wallace A (2019). p62-mediated selective autophagy endows virus-transformed cells with insusceptibility to DNA damage under oxidative stress. PLOS Pathog.

[84] Blazer LL, Neubig RR (2009). Small molecule protein-protein interaction inhibitors as CNS therapeutic agents: current progress and future hurdles. Neuropsychopharmacology.

[85] Tsuburaya N, Homma K, Higuchi T (2018). A small-molecule inhibitor of SOD1-Derlin-1 interaction ameliorates pathology in an ALS mouse model. Nat Commun.

