# Supplementary material for: Defective repair of topoisomerase I induced chromosomal damage in Huntington’s disease
Source: Cell Mol Life Sci. 2022 Feb 28;79(3):160. doi: 10.1007/s00018-022-04204-6 (PMC8882575; doi:10.1007/s00018-022-04204-6)
Supplement: Supplementary file 1 — Supplementary file1 (DOCX 4185 KB) [file 18_2022_4204_MOESM1_ESM.docx]

Supplementary information

**Title: Defective repair of topoisomerase 1 induced chromosomal damage in Huntington’s disease**

Nelma M. Palminha, Cleide dos Santos Souza, Jon Griffin, Chunyan Liao, Laura Ferraiuolo, Sherif F. El-Khamisy*

*Correspondence to: s.el-khamisy@sheffield.ac.uk

Supplementary data, materials and methods

Resampling of 53BP1 recruitment data

The BrdU incorporation assay indicated that ~15% of patient derived fibroblasts from healthy and HD patients were in S phase after a prolonged (23 hr) exposure to BrdU. By contrast, ~80% of MRC5 cells had incorporated BrdU after 23 hours (fig S1a and b). These results confirm that healthy and HD patient derived fibroblasts are slowly dividing and make it unlikely that the observed difference in 53BP1 recruitment is a cell cycle specific phenomenon. To formally test this we resampled the 53BP1 foci data 1000 times with replacement, selecting 85% of each dataset (the proportion in G1 phase) for each iteration. This simulated the effect of excluding the proportion of cells in S phase. For each resample we calculated the mean 53BP1 foci per cell and visualised the results as violin plots (fig S1c). CPT-induced 53BP1 foci per cell were significantly higher in healthy cells compared to either HD cell line. In addition, there was no difference in foci per cell between DMSO-treated cells.


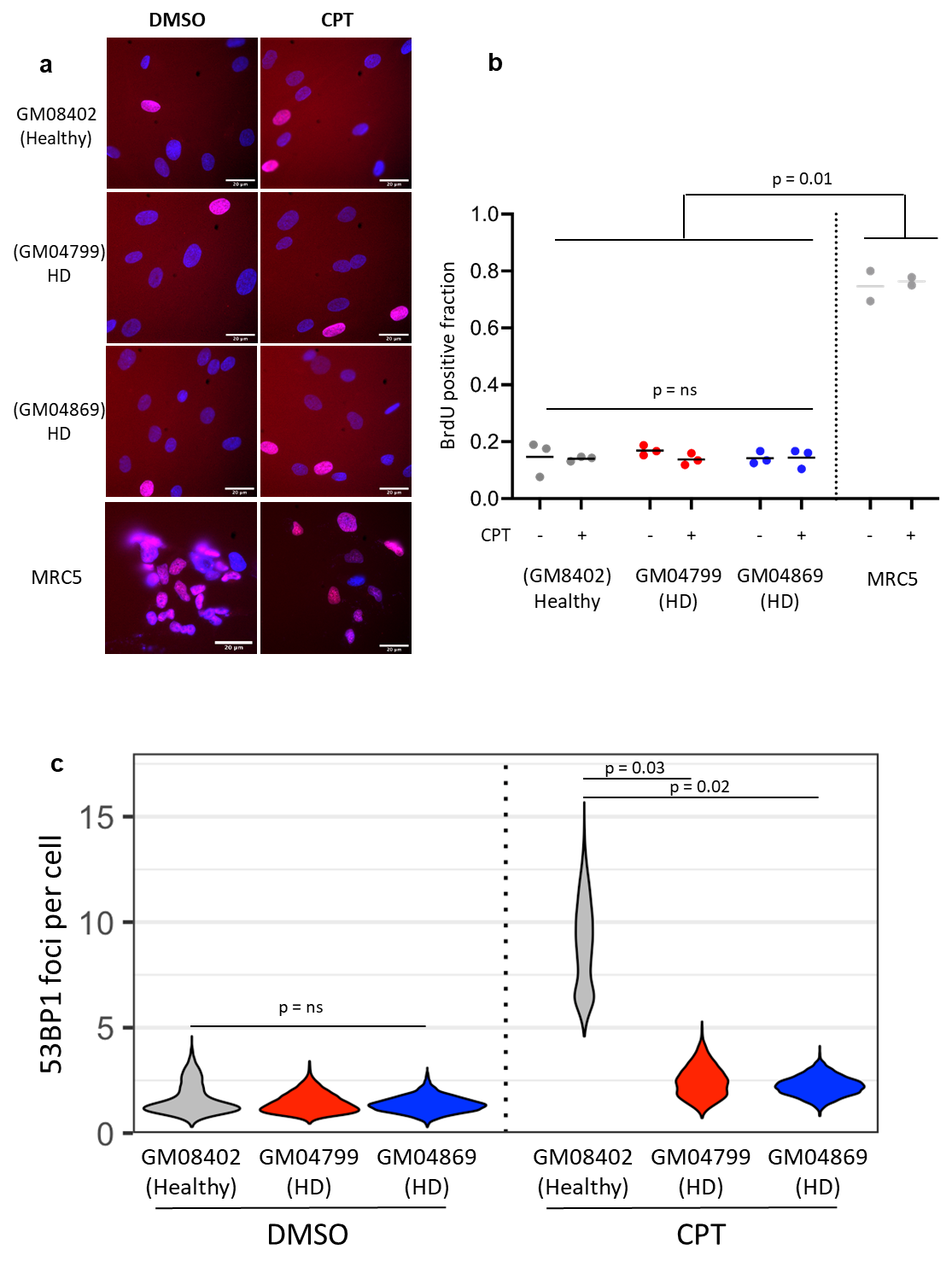


**Fig. S1 Deficient 53BP1 recruitment is not cell cycle specific**

**(a)** Representative images of primary skin fibroblasts from a healthy individual (GM08402) and two HD patients (GM04799 and GM04869). MRC5 cells were used as a positive control. Cells were incubated with BrdU for 23 hours and treated with DMSO or CPT for 1 hour then immunostained with anti-BrdU. Scale bar: 20 μm **(b)** The BrdU positive fraction for each cell type and condition. Each point represents the average BrdU positive fraction from one biological replicate. **(c)** Violin plot of resampled mean of 53BP1 foci per cell, adjusted to account for 15% of cells in S phase (see supplementary methods).


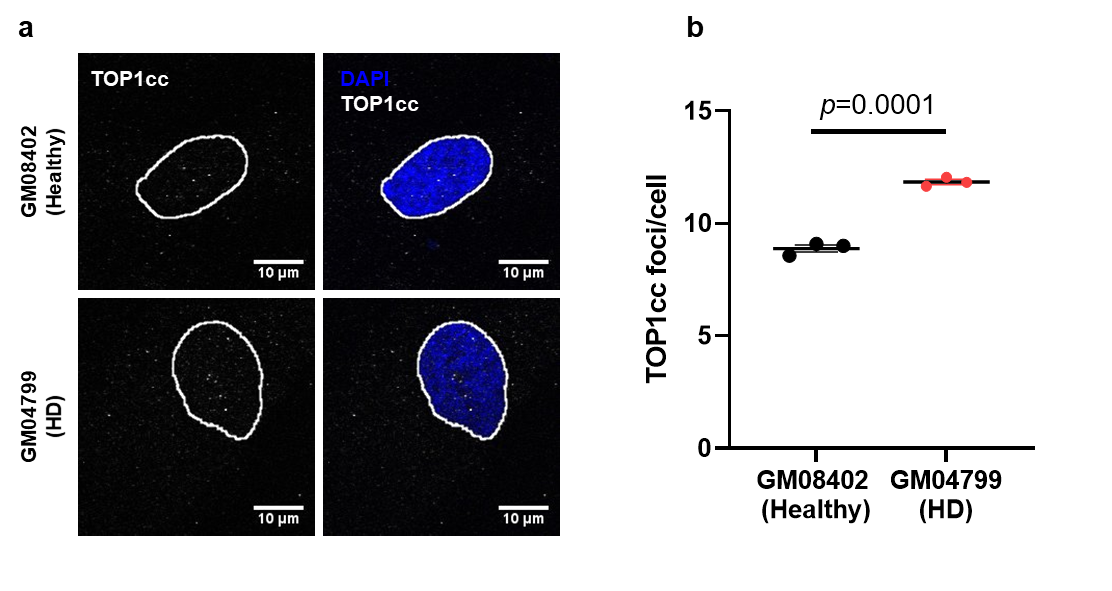


**Fig. S2** **HD patient-derived fibroblasts accumulate endogenous TOP1cc levels**

**(a)** Representative images of primary skin fibroblasts from a healthy individual (GM08402) and a HD patient (GM04799, 42 CAG) immunostained with an antibody against TOP1cc. Scale bar: 10 μm. **(b)** The number of TOP1cc foci per cell was quantified and analyzed by Student’s *t*-test. Data is presented as average of 3 independent experiments ±s.e.m.

**
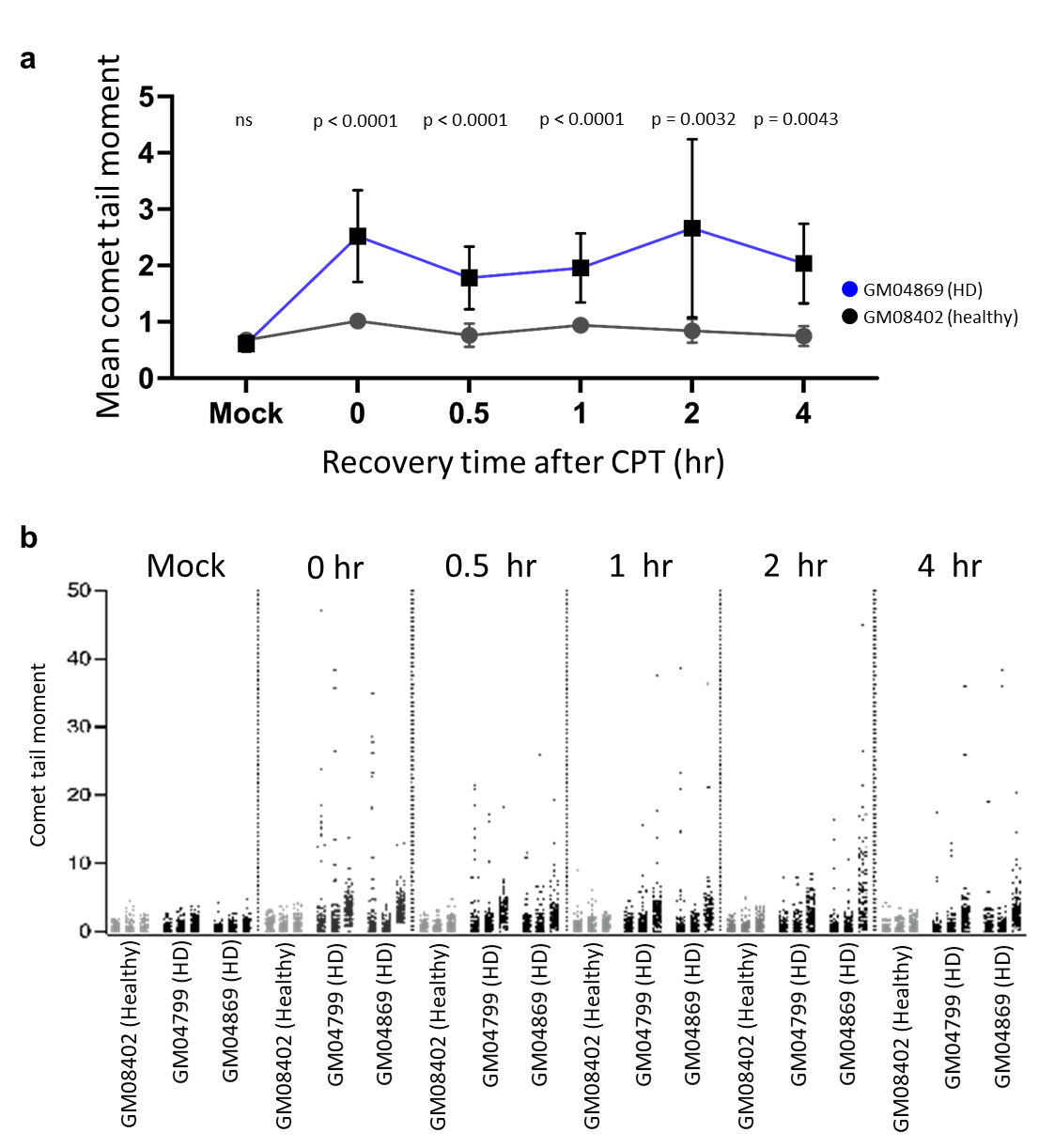
**

**Fig. S3 HD cells show higher levels of DNA damage after CPT**

**(a)** Related to figure 3c. Mean comet tail moments for a second independent patient-derived HD cell line (GM04869) under the same conditions as figure 3c.

**(b)** Comet tail moment data used to derive mean comet tail moments displayed in figure 3c and S3a. Each column represents one independent biological replicate of the cell line indicated.


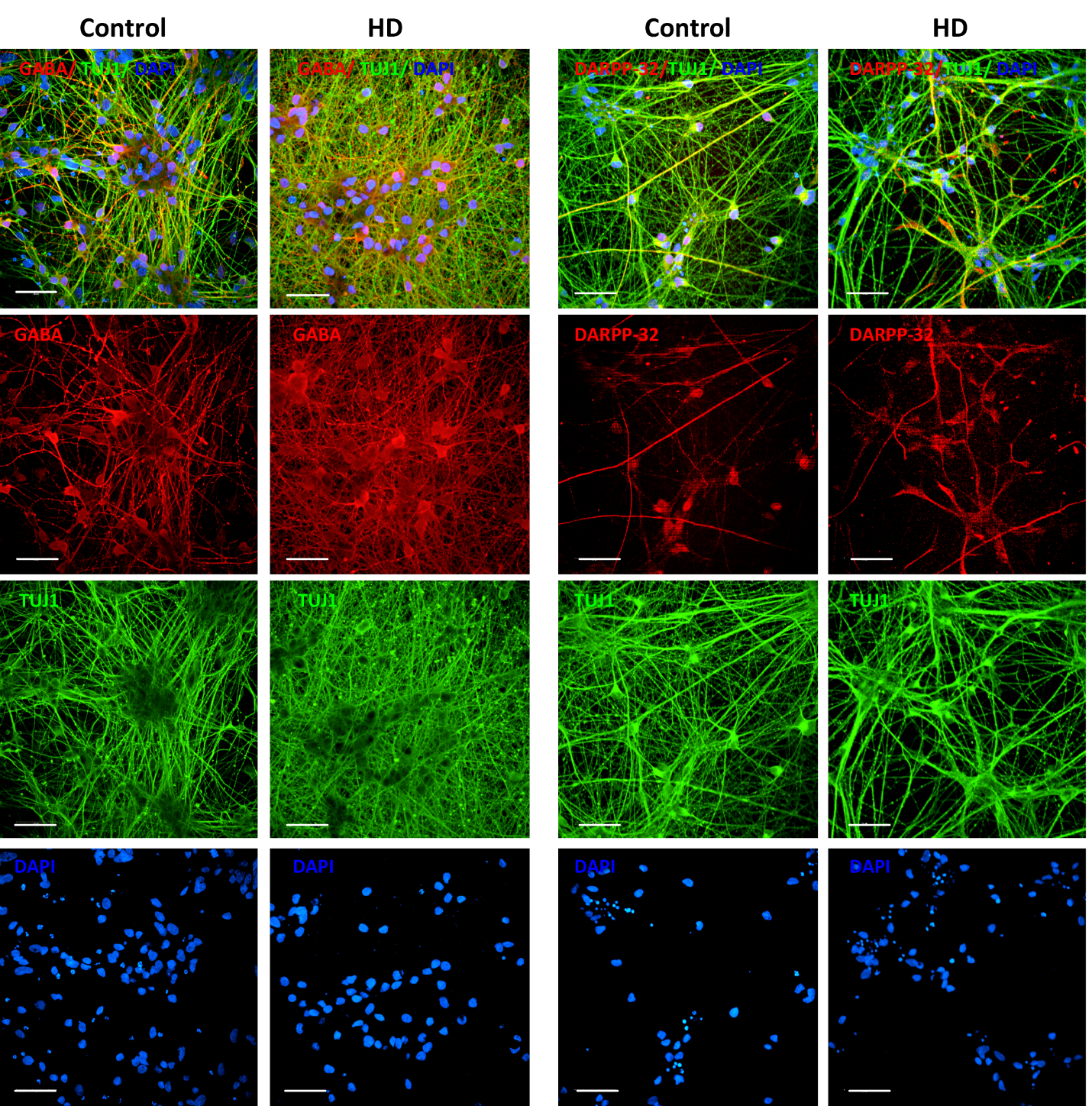


Fig. S4 Characterization of iPS-derived Striatal GABAergic neurons.

(a) Healthy and HD GABAergic neurons stained for the neuronal marker Beta III Tubulin (TUJ1, green) and GABAergic marker GABA (red). (b) Healthy and HD GABAergic neurons stained for DARPP32 (red) and Beta III Tubulin (Tuj1, green). Nuclei were counterstained with DAPI (blue); scale bar: 50 µm


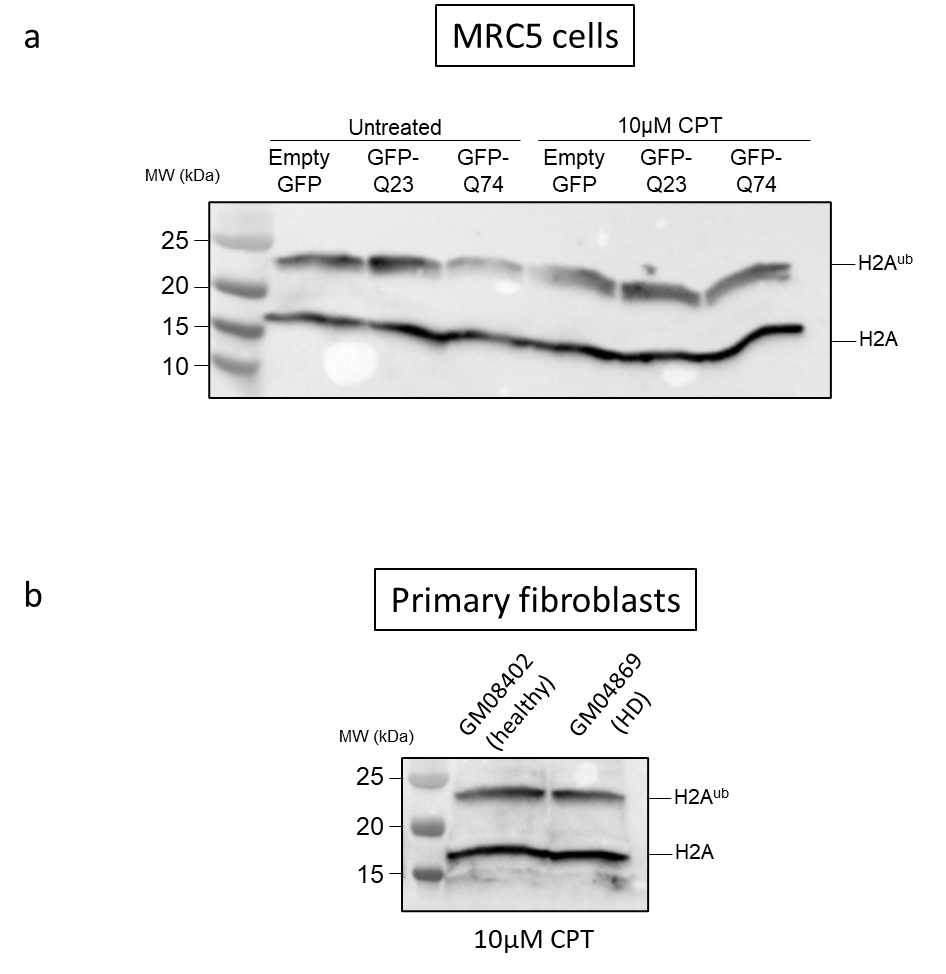


**Fig. S5 H2A ubiquitination in response to CPT**

**(a)** MRC5 cells were transiently transfected with empty vector GFP, GFP-Q23 or GFP-Q74 and treated with DMSO or 10 µM CPT for 1h. The cells were subjected to chromatin fractionation and analyzed by Western blot using H2A antibody that recognizes H2A and ubiquitinated H2A (H2A^ub^). **(b)** Healthy (GM08402) and HD (GM04869) primary fibroblasts were treated with 10 µM CPT for 1h. Chromatin-bound fractions were analyzed by Western blot using H2A antibody.

**Table S1 Primary cell lines**.

Details about HD diagnosis, number of CAG repeats, age at collection, gender, source and identifier are provided

| **Cell line** | **Diagnosis** | **(CAG)n** | **Age at collection (yr)** | **Gender** | **Source** | **Identifier** |
| --- | --- | --- | --- | --- | --- | --- |
| **Fibroblast** | | | | | | |
| GM08402 | Non HD | -- | 32 | Male | Coriell | RRID:CVCL_7485 |
| GM04869 | HD | 47 | 32 | Female | Coriell | RRID:CVCL_1I73 |
| GM04799 | HD | 42 | 47 | Male | Coriell | RRID:CVCL_Y887 |
| **iPSCs** | | | | | | |
| CS14iCTR | Non HD | -- | 30 | Female | Cedars-Sinai | RRID:CVCL_JK54 |
| GM23225 | HD | 68 | 20 | Female | Coriell | RRID:CVCL_F169 |
